# Flexible Unusual Ternary‐Component Graded‐Modulus Dielectric Films with High‐Density Capacitive Energy Storage

**DOI:** 10.1002/advs.75372

**Published:** 2026-04-24

**Authors:** Yi Gao, Xin‐Jie Wang, Lei Huang, Kun Xing, Baoquan Wan, Yan‐Hui Song, Jian‐Tao Wang, Li‐Juan Yin, Tong Liang, Yan Meng, Zunpeng Feng, Kai Huang, Ke Bi, Shao‐Long Zhong, Zhi‐Min Dang

**Affiliations:** ^1^ State Key Laboratory of Information Photonics and Optical Communications School of Physical Science and Technology Beijing University of Posts and Telecommunications Beijing China; ^2^ State Key Laboratory of Power System Operation and Control Department of Electrical Engineering Tsinghua University Beijing China; ^3^ Zhongyuan Electric Laboratory Xuchang Henan China

**Keywords:** breakdown strength, dielectric films, energy storage, hierarchical structure, multi‐component interfaces, trapping engineering

## Abstract

Polypropylene (PP) is widely used in film capacitors but is limited by an intrinsic trade‐off between dielectric permittivity and breakdown strength, capping energy density around 3 J cm^−^^3^. This limitation is overcome by introducing a liquid silicone rubber (LSR107) interphase and BaTiO_3_ (BT, diameter 5 nm) nanofiller into PP via one‐step melt extrusion, creating a ternary nanocomposite with a hierarchical interphase‐regulated structure. LSR107 selectively localizes in the amorphous/low‐crystallinity regions of PP and around BT‐rich regions, forming a compliant LSR107‐rich interphase that alleviates local electric‐field concentration, while BT provides a moderate permittivity increase. The optimized composite achieves a discharged energy density of 5.89 J cm^−^^3^ at 96% charge–discharge efficiency and a high breakdown field (∼640 kV/mm). Remarkably, it retains >480% elongation at break and shows no performance degradation over 1 00 000 charge–discharge cycles. This soft–hard interphase design circumvents the traditional permittivity–breakdown compromise, yielding a melt‐processable dielectric film compatible with roll‐to‐roll manufacturing for high‐frequency capacitors and flexible electronics.

## Graphical abstract

Hierarchical PP–LSR107–BaTiO_3_ nanocomposite films are fabricated by one‐step extrusion and stretching for roll‐to‐roll manufacture, combining high energy storage and processing flexibility. LSR107 surrounds BaTiO_3_‐rich regions, locating amorphous PP, decreasing defects, and introducing deep traps that homogenize the electric field and suppress charge transport. At 640 kV mm^−1^ the film displays an energy storage density of 5.89 J cm^−3^ with 96% efficiency and >480% elongation.

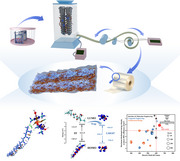

## 1 Introduction

In modern power electronics, film capacitors based on flexible polymer dielectric films (notably BOPP) are deployed on a massive scale (over 1 00 000 tons per year) owing to their low cost, high breakdown strength, and long‐term reliability [1, 2, 3]. These capacitors provide ultrafast charge–discharge capability and robust ripple‐current handling, making them ubiquitous in high‐power conversion systems [4, 5]. Moreover, polymer film capacitors feature extremely low equivalent series resistance and dielectric absorption, enabling efficient operation under high‐frequency switching conditions [6, 7]. In particular, Direct Current Link (DC‐link) capacitors in electric vehicle traction inverters must buffer high‐voltage direct current and handle large switching ripple currents, making them among the bulkiest components in an inverter [8, 9]. Meeting these demands requires maximizing volumetric energy density at nominal operating conditions (around 25°C) while maintaining stable dielectric performance under continuous high field cycling and ripple stress [10, 11, 12]. For example, BOPP‐based film capacitors can withstand more than 10^4^ consecutive charge–discharge cycles at 400 kV/mm with minimal degradation, reflecting the high cyclic endurance required of DC‐link films [13, 14, 15]. However, the push toward miniaturization in electric vehicles and other applications increases pressure on capacitor size.

Polymer film capacitors underpin DC‐link power modules because they combine rapid discharge capability, low dielectric loss, and high breakdown strength. Commercial systems still rely on biaxially oriented polypropylene (BOPP) films with a relative permittivity of only about 2.2 at room temperature [16, 17]. For an ideal linear dielectric, the stored energy density follows 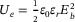 so a limited ε_*r*_ directly constrains *U_e_* even when*E_b_* is high [18, 19]. BOPP film can reach breakdown strengths on the order of 400 to 600 kV/mm, yet commercial BOPP films provide only about 2.9 J cm^−^^3^ at 550 kV mm ^−^^1^, leaving very limited volumetric headroom for further miniaturization without a new materials paradigm [13]. Crucially, capacitor‐grade PP films are produced by high‐ratio roll‐to‐roll drawing to achieve micrometre‐scale thickness, so any modified PP dielectric must retain very high tensile strain during uniaxial stretching to enable thinning without tearing or defect formation [20, 21].

In recent years, a broad set of modification strategies on PP has been explored to overcome the performance ceiling of polymer dielectrics for electrostatic energy storage, including composites with high permittivity inorganic fillers, incorporation of polar polymers or dipolar moieties, multilayer heterostructures, and charge trap engineering [22, 24]. Ceramic nanoparticles (such as BaTiO_3_, SrTiO_3_) can substantially increase permittivity, but the large variation in dielectric permittivity between the ceramic phase and the polymer matrix tends to generate strong electric field concentrations at interfaces that sharply reduce breakdown strength, while high filler loadings notably increase dielectric loss and introduce interfacial defects that undermine film quality and reliability [25, 26, 27, 28, 29]. All organic blending improves compatibility and manufacturability, yet it often struggles to raise discharged energy density and efficiency simultaneously. Polar functionalization can increase polarization, but it often amplifies dipolar relaxation and leakage, and a high fraction of shallow traps can intensify dielectric loss under high fields [30, 31, 32]. Multilayer designs can redistribute the electric field and introduce micro interfaces that impede carrier migration, but the additional interfaces also create discontinuities and failure‐prone weak planes [33, 34, 35, 36]. Trap engineering via inorganic barriers or electron affinity dopants can suppress conduction, yet phase separation and reduced flexibility complicate large area film formation, and trap levels may still permit detrapping under strong fields [18, 37]. This field, therefore requires a specific modified polypropylene structural strategy that translates these general notions of interfacial polarization and trap engineering into a testable, mechanism‐based design rationale.

In this work, a scalable hierarchical PP‐LSR107‐BaTiO_3_ ternary‐component platform is introduced that combines nanoscale interfacial design with industrial melt extrusion to address these limitations. First, a continuous one‐step melt extrusion process with in‐line stretching is implemented to produce thin PP‐LSR107‐BaTiO_3_ films, thereby eliminating solvent casting and intermediate multi‐step compounding. Second, the components self‐organize during flow into a hierarchical morphology, in which LSR107 forms a compliant LSR107‐rich interphase within the amorphous regions of PP and around BT‐rich regions, thereby homogenizing local electric fields and suppressing interfacial charge injection. Third, LSR107 provides deep trap engineering that broadens the distribution of energetically deep trapping sites in the composite, thereby reducing high‐field charge transport by approximately one order of magnitude. By coupling nanoscale interface engineering with mesoscale phase morphology control, the permittivity, breakdown strength, and dielectric loss are no longer passively constrained by the same tradeoffs. As a result, the composite film delivers markedly enhanced discharged energy density together with high charge–discharge efficiency under technologically relevant electric fields, establishing a new design paradigm for polypropylene‐based energy‐storage dielectrics.

## 2 Results and Discussion

### 2.1 Continuous Fabrication and Hierarchical Structure

**FIGURE 1:**
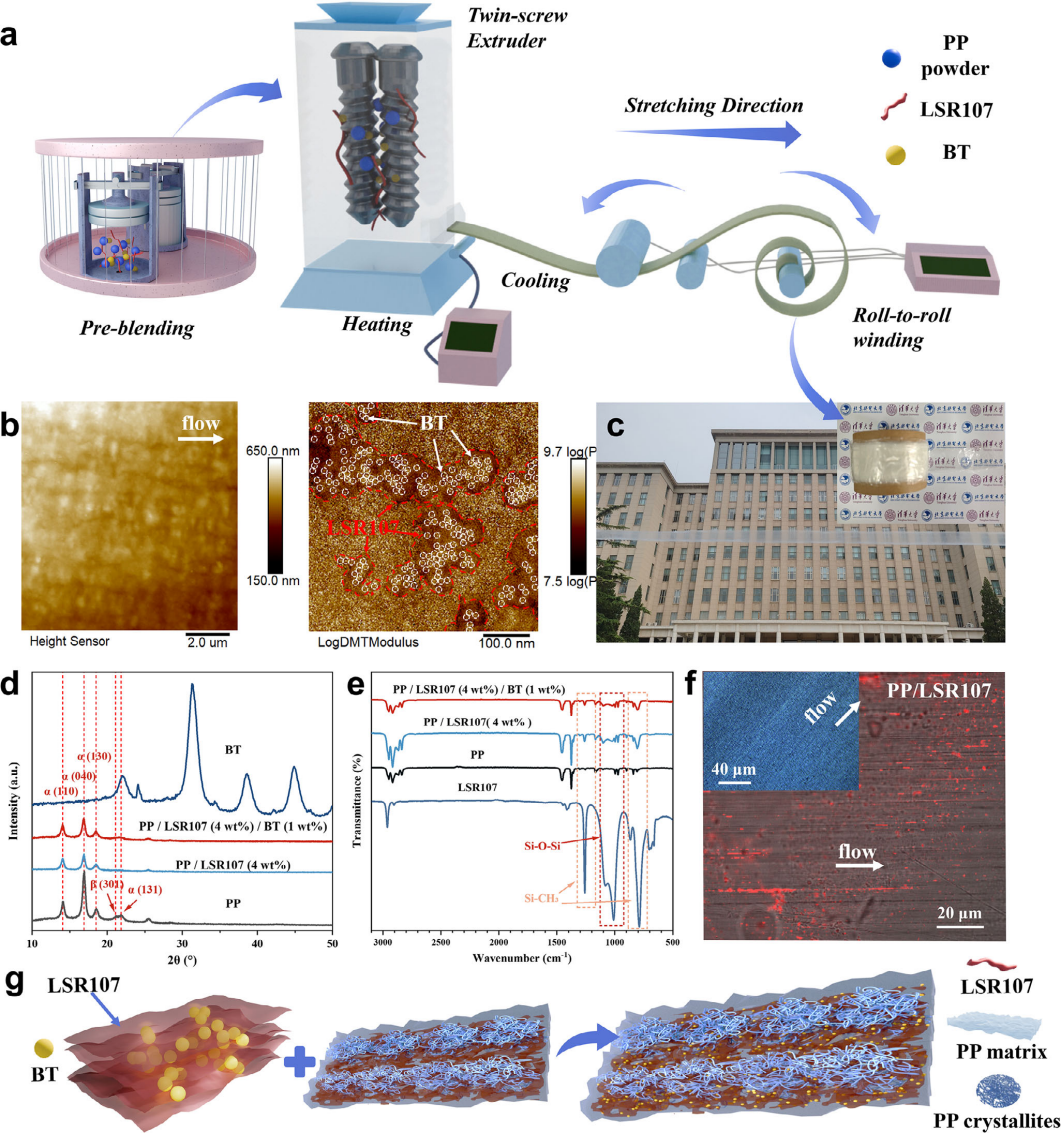
Continuous one‐step fabrication and hierarchical structure of polypropylene (PP)/liquid silicone rubber (LSR107)/barium titanate (BT) composite films. (a) Schematic of the continuous melt‐processing route including premixing, twin‐screw extrusion, uniaxial stretching, cooling, and roll‐to‐roll winding. (b) PeakForce tapping AFM modulus maps of a PP/4.0 wt.% LSR107/1.0 wt.% BT film, showing aligned shish‐kebab crystalline features along the stretching direction (left) and nanoscale heterogeneity associated with LSR107‐rich domains and BT‐rich regions (right). (c) Photograph of a meter‐scale flexible composite film (10 cm in width). (d) X‐ray diffraction (XRD) patterns of PP and composites, showing preserved PP crystal structure and BT reflections. (e) FTIR spectra confirming the chemical integrity of LSR107 in PP (Si‐O‐Si and Si‐CH_3_ vibrations) with no new covalent bond formation. (f) Polarized optical microscopy (POM, top) and confocal laser scanning microscopy (CLSM, bottom; Nile Red staining) images revealing oriented PP crystallites and LSR107‐rich domains along the stretching direction. (g) Schematic of the hierarchical architecture, in which LSR107 is preferentially localized in amorphous/low‐crystallinity PP regions and around BT‐rich regions, forming compliant LSR107‐rich interphases that partially encapsulate local BT clusters.

The PP‐LSR107‐BT composite film was fabricated in a one‐step, continuous extrusion process (Figure 1a) that combines melt‐compounding with drawing to produce meter‐scale films of uniform thickness (Figure 1c). This continuous approach eliminates intermediate pre‐processing, directly converting raw materials into functional dielectric films through synchronized internal mixing and uniaxial stretching. The resultant free‐standing film (4 wt.% LSR107, 1 wt.% BT) exhibits macroscopic uniformity over meter‐scale dimensions (10 cm width, Figure 1c), demonstrating compatibility with industrial roll‐to‐roll production, with the flexibility required for winding and handling in capacitor manufacturing [38].

Crystallinity of the PP matrix and incorporation of BT were examined by X‐ray diffraction (XRD, Figure 1d and Figure S4). The XRD pattern of the composite shows the characteristic diffraction peaks of α‐polypropylene, appearing at 2θ about 14.0°, 16.8°, 18.5°, 21.7°, and 25.4° (indexed to the (110), (040), (130), (131), and (060) planes, respectively). These reflections match neat iPP, indicating that PP remains semi‐crystalline and largely in the α phase [39]. Importantly, the addition of 4 wt.% LSR107 does not disrupt the PP crystallites, and the same set of characteristic peaks is preserved in the PP/LSR107 binary blend's diffractogram. Upon introduction of BT, additional Bragg peaks emerge at higher angles (Figure 1d). A strong reflection at 2θ about 31° corresponds to the (110) plane of perovskite BaTiO_3_. As further clarified by the separate characterization of the as‐synthesized BT nanoparticles (Figure S3), the BT powder is more appropriately described as predominantly cubic or pseudo‐cubic, with trace carbonate‐related surface species. Accordingly, the BT‐related reflections in Figure 1d are assigned to crystalline perovskite BaTiO_3_ rather than a well‐developed tetragonal phase. Importantly, no new processing‐induced diffraction peaks or obvious peak shifts are observed in the composite patterns relative to the individual constituents [40]. The coexistence of distinct PP and BT reflections, together with the retention of the characteristic α‐PP peaks after the addition of LSR107 and BT, implies that the polymer and BT are physically mixed with minimal chemical interaction, which is beneficial for maintaining the excellent insulating properties and mechanical strength of the polymer host.

Fourier‐transform infrared (FT‐IR) spectroscopy was used to assess the chemical composition of the composite and potential interfacial interactions (Figure 1e). The spectra show superimposed bands from the PP matrix and the LSR107 phase, indicating that both components remain chemically intact after processing. PP characteristic bands appear at 2950 and 2915 cm^−1^ for methyl and methylene C‐H stretching, 1455 cm^−1^ for CH_2_ bending, and 1377 cm^−1^ for CH_3_ symmetric bending. LSR107 related bands are also evident in the fingerprint region, including a Si─O─Si stretching band at 900 to 1010 cm^−1^ and Si‐CH_3_ deformation or rocking bands near 800 cm^−1^ and 1200 to 1300 cm^−1^. These features match the spectrum of neat LSR107 (Figure S5), consistent with its polydimethylsiloxane backbone [41, 42]. The retention of distinct PP and LSR107 bands suggests that no new covalent bonds form during compounding, and the ternary system remains physically blended. Furthermore, the BT filler (an inorganic oxide) does not show direct IR‐active vibrations in this range, but its indirect presence can be inferred from any changes in the polymer peaks. Here, only minor intensity changes are noted, suggesting good compatibility and possibly weak interfacial bonding (e.g., via van der Waals) between PP and LSR107. Overall, the FT‐IR analysis confirms that the composite's internal structure consists of original constituents (PP, LSR107, BT) combined without chemical degradation. PP retains its backbone (no oxidative carbonyl peak 1720 cm^−1^ was detected), and LSR107 retains its siloxane network (no significant 2160 cm^−1^ Si─H signal is seen).

Atomic force microscopy (AFM) modulus mapping of the film's surface (Figure 1b) reveals a heterogeneous yet well‐integrated microstructure, the local Derjaguin‐Muller‐Toporov (DMT) modulus spans from 10 MPa (rubbery domains) to 1GPa (stiffer crystalline domains). Such a broad stiffness distribution reflects coexisting soft LSR107‐rich regions and stiff domains associated with PP crystallites and 5 nm BT inclusions [43]. Notably, LSR107 is mainly distributed within the amorphous and low‐crystallinity regions of the PP matrix rather than within the highly crystalline PP domains. After Nile Red staining, the LSR107‐rich regions appear as elongated domains aligned along the drawing direction (Figure 1f and Figures S6–S7). This multiscale soft–hard co‐distribution is expected to facilitate stress transfer and alleviate local strain and electric‐field concentration in the composite.

The extruded films (Figure 1f and Figure S6) directly visualize phase morphology after selective Nile Red staining of the LSR107 phase. The POM micrograph (Figure 1f, inset, oriented 45° to the draw direction) reveals the highly oriented shish‐kebab PP crystalline structures, while the CLSM image (Figure 1f main panel) shows bright red‐fluorescent LSR107‐rich domains confined between these PP crystallites. In the binary PP‐LSR107 blend, the LSR107 phase is clearly aligned along the drawing direction, parallel to the PP crystalline lamellae. This indicates a co‐continuous minor‐phase morphology induced by the strong extensional flow during melt processing. Supplementary confocal images (Figure S6, bright‐field vs. fluorescence) further confirm that the red‐stained LSR107 is concentrated in the amorphous or low‐crystallinity regions of PP, running in continuous paths between the oriented PP crystallites.

In the ternary LSR107/BT/PP composite, the same structural motif is preserved, and importantly, the BT nanoparticles are found to be selectively located within or around the LSR107 fibrils rather than dispersed randomly in the PP matrix. Consistent with AFM (Figure 1b), TEM images(Figure S8), and CLSM images (Figure 1f and Figure S6) indicate that BT is present mainly as local nanoscale clusters, while LSR107 forms broader soft interphase domains around these BT‐rich regions. Therefore, the morphology is more appropriately described as partial or broad encapsulation of local BT clusters by LSR107‐rich domains. For comparison, the PP/BT (1 wt.%) binary composite shows local BT clustering without the broader compliant LSR107‐rich interphase, as evidenced by the AFM, TEM, and SEM/EDS results in Figure S9. This preferential localization is illustrated schematically in Figure 1g and Figure S7, where local BT clusters are embedded in compliant LSR107‐rich domains that are spatially confined within the continuous PP matrix. Such a hierarchical morphology is expected to mitigate direct BT–BT contact, buffer filler–matrix mismatch, and reduce local mechanical and electrical stress concentration.

The schematic (Figure 1g) highlights the material's hierarchical structure, (i) at the nanoscale, BT exists mainly as local BT clusters or BT‐rich regions surrounded by a compliant LSR107‐rich interphase; (ii) at the microscale, PP these LSR107‐buffered BT‐rich regions are aligned within the minor phase of the PP matrix; (iii) at the macroscale, the film exhibits a mutually complementary hierarchical architecture, comprising alternating soft and hard phase regions through the thickness, all consolidated via melt‐processing [44, 45]. The structure‐property correlation is clear. By distributing the stiff BT particles within pliable LSR107 domains that are in turn anchored in a strong PP matrix, the composite can simultaneously achieve high dielectric response and excellent mechanical/electrical integrity. The PP crystalline framework provides high tensile strength and baseline dielectric strength, the LSR107 elastomer absorbs mechanical and electrical stress (preventing premature failure around the ceramics), and the BT delivers enhanced polarizability for greater permittivity. This hierarchical architecture provides a scalable route to improved dielectric performance through coupled component selection and flow‐induced morphology control.

A scalable methodology is established for producing hierarchically structured PP‐LSR107‐BT composites, bridging molecular design with industrial processing. The continuous one‐step fabrication integrates pre‐blending and uniaxial stretching, allowing direct conversion of raw materials into meter‐scale films at speeds compatible with roll‐to‐roll production. By circumventing solvent casting and intermediate compounding, this approach markedly streamlines the manufacturing process and lowers capital expenditure relative to multi‐component co‐extrusion.

### 2.2 Synergistic Dielectric Energy Storage in Ternary Composites

**FIGURE 2:**
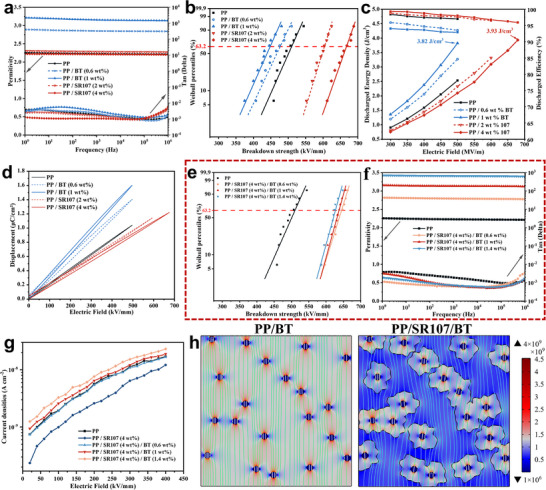
Dielectric properties and synergistic energy‐storage performance of PP/LSR107/BT composites. (a) Frequency‐dependent relative permittivity (*ε_r_*) of PP‐based composites with varied BT and LSR107 contents. (b) Weibull breakdown‐strength distributions of PP/BT and PP/LSR107 binary composites. (c) Discharged energy density as a function of filler content at different electric fields. (d) Representative D‐E loops at the maximum applied field and corresponding discharged energy density‐electric field characteristics for the binary systems. (e) Frequency‐dependent *ε_r_* and loss tangent (*tan δ*) of ternary PP/LSR107/BT composites. (f) Weibull breakdown‐strength distributions of neat PP and ternary composites. (g) Leakage current density as a function of electric field for neat PP, PP/BT (1 wt.%), PP/LSR107 (4 wt.%), and PP/LSR107/BT (1 wt.%); the inset shows an enlarged view of the high‐field region. h) Finite‐element simulation of local electric‐field distribution around a BT particle in PP (binary) and around an idealized BT‐rich inclusion surrounded by an LSR107 interphase (ternary) under an applied field.

PP exhibits a low relative permittivity (≈2.2 at 100 Hz), which is one of the factors limiting its energy storage density. Consequently, incorporating a small fraction of BT noticeably raises the dielectric constant; for instance, with 1 wt.% BT, the composite's *ε_r_* increases to 3.2 (Figure 2a), indicating enhanced polarization. In contrast, adding LSR107 up to 4 wt.% yields little change in *ε*_r_, which remains about 2.3. For dielectric energy storage, however, the discharged energy density *U_d_* is determined by both the permittivity and the maximum electric field strength. In fact, *U_d_* corresponds to the area enclosed by the discharge branch of the D–E loop (i.e. *U_d_* = ∫*EdD*). As a result, neither single filler composite yields a dramatic increase in discharged energy density. Neat PP stores about 2.53 J cm^−3^ at breakdown, PP with 1 wt.% BT reaches about 3.82 J cm^−3^ (Figure 2c,d), and PP with 4 wt.% LSR107 attains about 3.93 J cm^−3^ [23]. These incremental gains reflect the classic trade‐off between permittivity and breakdown strength, where the higher *ε*_r_ from BT is limited by a reduced breakdown strength (Figure 2b), while the improved breakdown from LSR107 is limited by its unchanged permittivity (see also Figure S12) [23].

To overcome this trade‐off, the ternary PP/LSR107/BT composite is designed to increase *ε*_r_ while retaining a high breakdown field. Indeed, as shown in Figure 2e,f, the ternary films achieve a moderate permittivity increase due to BT while maintaining high breakdown strength enabled by LSR107. Consequently, the PP/LSR107/BT composites exhibit higher discharged energy density than either binary counterpart. For example, the PP/4.0 wt.% LSR107/1.0 wt.% BT sample shows a significantly higher dielectric constant than neat PP, with only a slight reduction in breakdown strength relative to PP/4.0 wt.% LSR107.

Figure 2b,f present Weibull breakdown strength data for these materials, and Figure 2g compares their leakage current densities. These measurements underscore the excellent insulating nature of PP and how each additive affects it. Breakdown data follow a two‐parameter Weibull distribution

(1) $$P\left(E\right)=1-\exp\left[-{\left(\frac{E}{\alpha }\right)}^{\beta }\right]$$

where *α* is the characteristic field corresponding to 63.2% failure probability [46]. Neat PP has a characteristic breakdown field of *α* about 511 kV mm^−1^, and adding 4 wt.% LSR107 raises it to  667 kV mm^−1^, representing a significant improvement in dielectric strength. The PP/BT composite breaks down at lower fields than neat PP, and its leakage current climbs more rapidly with increasing field. Crucially, the ternary composite with both LSR107 and BT nearly preserves the high breakdown strength of PP/4 wt.% LSR107, which is within about 5–10% of the value for the only LSR107 composite (Figure 2f). This indicates that the LSR107 interfacial layer effectively buffers the field intensification that BT would otherwise introduce. Neat PP accordingly shows extremely low electrical leakage (below 1.2 × 10^−^^8^ A/cm^2^ up to 350 kV/mm), and adding 4 wt.% LSR107 also lowers the leakage current at intermediate fields (at 300 kV/mm, leakage is 30% lower than in pure PP). Meanwhile, BT‐filled samples exhibit an earlier onset of conduction, with leakage‐field curves rising steeply at lower fields than in neat PP or PP/LSR107. By including LSR107 alongside BT, the ternary composite avoids this early leakage surge and retains insulating behavior comparable to PP with LSR107 alone up to the breakdown threshold.

The inset in Figure 2g further highlights the high‐field region, where the divergence between PP/BT and PP/LSR107/BT becomes more evident. To clarify the charge‐transport behavior, the leakage‐current data were further analyzed using Schottky emission, Poole–Frenkel emission, space‐charge‐limited conduction (SCLC), and hopping models (Figure S14) [11, 47]. Although the Schottky and Poole–Frenkel plots show partial linearity, the fitted parameters are not physically reasonable for the present PP‐based films, and the log(J)–log(E) curves do not exhibit a sufficiently broad and stable region characteristic of trap‐free SCLC. By comparison, the hopping representation provides the most self‐consistent description of the experimental data. Therefore, the leakage‐current behavior of the present PP‐based films is considered to be mainly consistent with trap‐assisted hopping‐like conduction. In this framework, BT alone promotes earlier conduction because of interfacial field concentration, whereas LSR107 suppresses carrier transport by introducing a more insulating interphase together with deeper trapping states, thereby mitigating the leakage increase caused by BT in the ternary composite.

The role of LSR107 is 2 fold: it improves the insulating quality of the PP‐rich amorphous regions, and in the ternary system, it further forms a compliant LSR107‐rich interphase around BT‐rich regions, thereby buffering BT‐induced local field intensification and suppressing interfacial charge transport. In a PP/BT composite without LSR107, the high‐permittivity BT filler tends to concentrate the electric field at its interfaces, leading to premature breakdown and elevated leakage. By contrast, in the ternary blend, LSR107 is preferentially localized around local BT‐rich regions (Figure 1g), forming an LSR107‐rich interphase that reduces these field intensifications and delays dielectric failure. This approach is analogous to reported core–shell filler strategies, where an insulating nanoscale coating on ceramic inclusions yields composites with both high permittivity and high breakdown strength. Finite‐element electrostatic simulations (Figure 2h) corroborate this mechanism. Because the dielectric response of nanosized/fine‐grained BaTiO_3_ is strongly size and structure‐dependent, the simulation input for BT was corrected using the experimentally measured dielectric constant of a pressed‐and‐sintered BT pellet (Figure S10). Specifically, the PP/LSR107/BT composite shows much less field distortion around BT than a BT‐filled PP without LSR107. Consequently, with LSR107 present, interfacial leakage at BT surfaces is minimized, and the breakdown strength remains nearly as high as in the only LSR107 system.

Overall, the one‐step blending of PP, LSR107, and BT produces a hierarchical microstructure that directly translates into these electrical improvements. Neat PP provides the baseline of excellent insulation and low dielectric loss, which is preserved and even slightly enhanced by LSR107, while BT offers a moderate permittivity boost. Crucially, LSR107 enables the inclusion of BT without sacrificing dielectric strength or causing excessive leakage. The resulting ternary composite at low loading contents achieves a balanced performance by combining increased permittivity with a high breakdown field and low leakage current, which yields a significantly improved energy storage density relative to unfilled PP and validates the design strategy.

### 2.3 Interfacial Trap Engineering and Field Homogenization Enabled by LSR107

**FIGURE 3:**
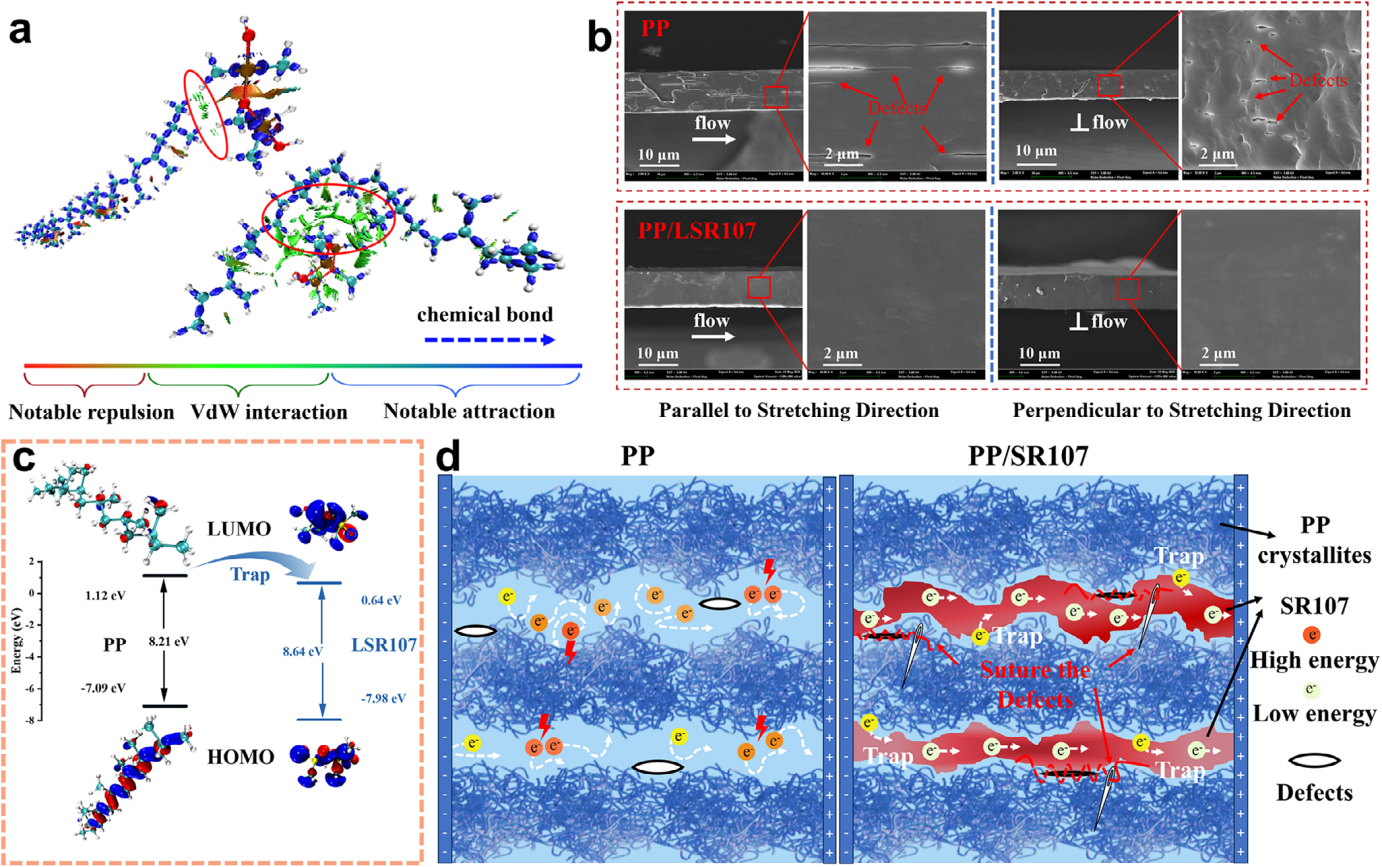
Interfacial trap engineering and field homogenization enabled by LSR107. (a) Independent gradient model‐based interaction region indicator (IRI) analysis indicating noncovalent interactions between LSR107 hydroxyl groups (‐OH) and PP methyl groups (‐CH_3_) (green isosurfaces). (b) Cross‐sectional SEM images of neat PP and PP/LSR107 (4.0 wt.%) films viewed parallel and perpendicular to the stretching direction. (c) Calculated frontier molecular orbitals (HOMO/LUMO) and band gaps of PP and LSR107, indicating deeper electronic states for LSR107. (d) Schematic illustration of the interphase‐regulated mechanism in the PP/LSR107/BT ternary composite. The compliant LSR107‐rich interphase around BT‐rich regions helps reduce direct filler–filler contact, fills local free‐volume/void‐like defects in the amorphous PP regions, and mitigates local stress and electric‐field concentration.

Interfacial interactions were assessed by interaction region indicator (IRI) analysis (Figure 3a), revealing noncovalent van der Waals contacts between LSR107 hydroxyl groups (‐OH) and PP methyl groups (‐CH_3_) [48, 49, 50, 51, 52]. Corroborating the CLSM characterization in Figure 1f, LSR107 molecules intercalate into the amorphous PP matrix and interact with the polymer mainly through weak van der Waals forces (green isosurfaces), with no evidence of covalent bonding as shown in Figure 1e. This indicates that LSR107 preferentially resides in the low‐crystallinity (amorphous) regions of the film, effectively filling free volume, which has an effect on electric insulation. Accordingly, LSR107 incorporation refines the PP morphology and reduces void volume (Figures S6 and S7). By occupying interstitial sites and sealing morphological defects, LSR107 passivates weak points, yielding a compact morphology and reducing local field enhancement.

The microstructural evolution mirrors the macroscopic dielectric trends. Neat PP exhibits a porous, void‐rich fracture surface (Figure 3b). LSR107 addition (4 wt.%) substantially eliminated these voids, yielding a denser morphology [53]. Crucially, BT incorporation (1 wt.%) further refined the microstructure, generating a homogeneous, defect‐free morphology without introducing new voids, irrespective of observation direction (parallel or perpendicular to stretching) (Figure S15). Elemental mapping demonstrated a homogeneous dispersion of BT within the ternary composite (Figure S1), validating effective interfacial integration.

The LUMO and HOMO energy levels (Figure 3c) indicate that LSR107 possesses a significantly larger band gap (8.64 eV) than PP (8.21 eV). This wider band gap aligns with the results obtained from UV–vis spectroscopy (Figure S16). Both the LSR107 HOMO and LUMO are shifted to lower energy (deeper) relative to PP. This means electrons in PP require more energy to reach the LSR107 levels. The deeper alignment of both valence and conduction band edges in LSR107 reflects its stronger insulating character. Because LSR107's LUMO lies well below the LUMO of PP, electrons injected into the PP conduction band are preferentially captured (trapped) by LSR107. In other words, LSR107 provides deep trap states for electrons. This trap‐assisted capture reduces the population of mobile carriers in the composite. Indeed, materials with a more compact microstructure (i.e., fewer structural defects) and deeper trap energy levels typically exhibit lower electrical conductivity and higher dielectric breakdown strength. This trap effect complements the morphological confinement revealed in Figure 1, where LSR107 resides at PP‐BT interfaces, and the field redistribution shown in Figure 2h, together isolating charges both spatially and energetically. The combined mechanisms delay charge injection, suppress space‐charge accumulation, homogenize local electric fields, and ultimately elevate breakdown strength, consistent with theoretical and experimental evidence that deeper and denser traps enhance dielectric reliability.

Figure 3d illustrates the full interfacial mechanism. LSR107 molecules fill the low‐crystallinity and amorphous regions at polymer/filler interfaces, acting like a ‘suture’ that bridges local free‐volume/void‐like defects. These LSR107 domains create deep trap sites (yellow symbols) that immobilize charge carriers. As a result, space‐charge accumulation is curtailed, and local electric‐field peaks are leveled. This trap‐mediated field homogenization effect has been shown to improve dielectric breakdown, as the introduction of more numerous and deeper traps can enhance breakdown strength and volume resistivity. Thus, LSR107 enhances dielectric reliability by simultaneously refining the microstructure (through defect filling and interfacial densification) and boosting trap density, thereby producing a synergistic structure‐to‐function effect corroborated by the analyses in Figure 3.

### 2.4 Scalable Manufacturing and Application Performance

**FIGURE 4:**
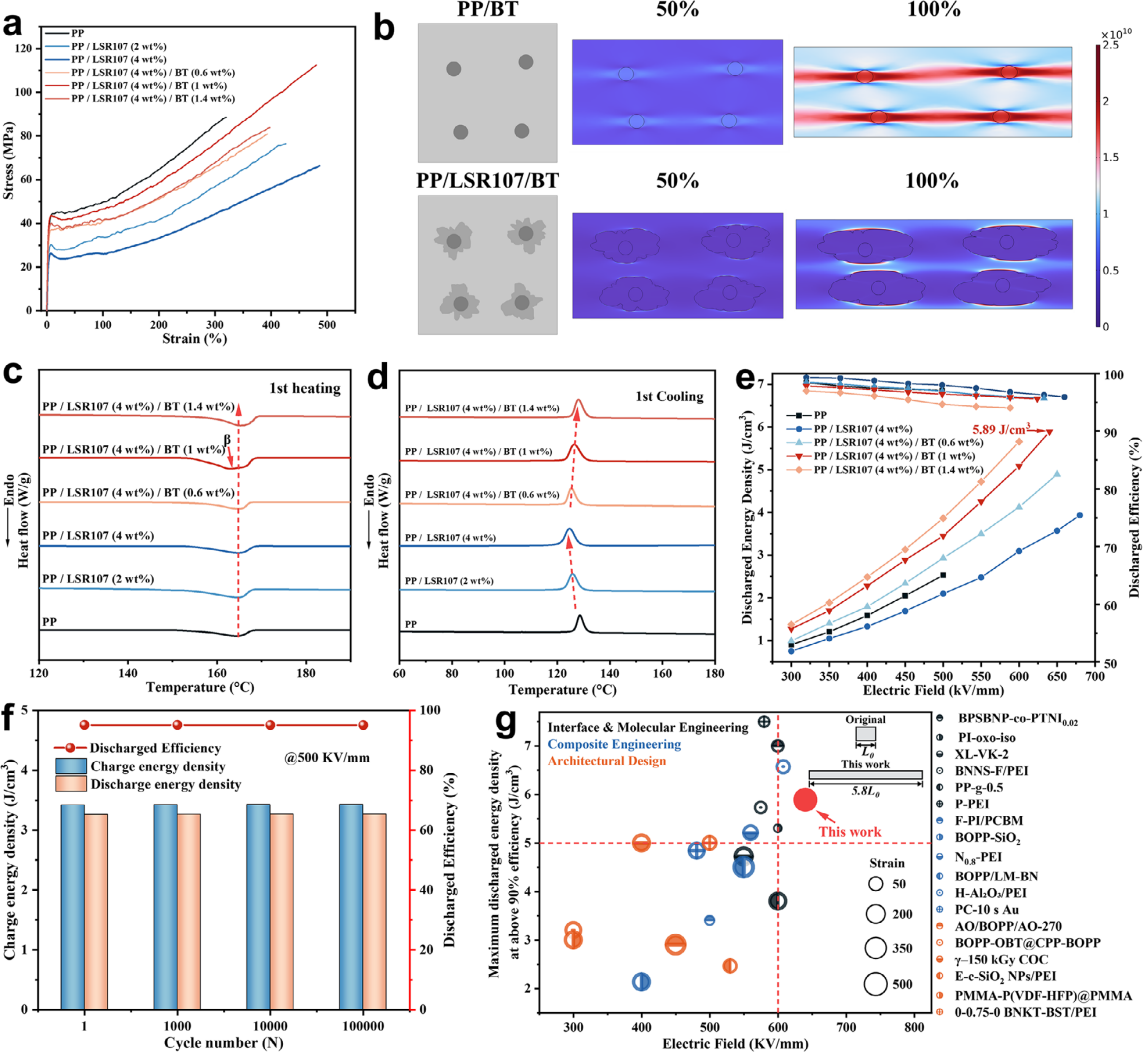
Mechanical robustness, thermal behavior, and application‐relevant performance. (a) Stress–strain curves of neat PP and composites, showing increased tensile strength (43.66 MPa vs. 26.15 MPa for PP/4.0 wt.% LSR107) while retaining high ductility (strain at break exceeding 400%). (b) Finite‐element analysis of stress redistribution in stretched PP composites with and without an LSR107 interphase. (c) Differential scanning calorimetry (DSC) heating (melting) curves (5°C min^−1^). (d) DSC cooling (crystallization) curves (5°C min^−1^). (e) Discharged energy density and charge‐discharge efficiency of PP films with varying LSR107 and BT contents. (f) Cycling stability of the optimized film at 500 kV mm^−1^ and 100 Hz for 10^5^ cycles (charge–discharge energy and efficiency). (g) Benchmarking of discharged energy density, efficiency, and mechanical performance against representative state‐of‐the‐art polymer dielectric strategies (bubble size denotes elongation at break).

Mechanical testing (Figure 4a) demonstrates that adding LSR107 and BT improves the tensile performance of the polymer. Relative to neat PP, PP/4 wt.% LSR107 maintains the tensile strength at 26.15 MPa while raising the strain at break from 320% to 480%, and subsequent incorporation of 1 wt.% BT increases the tensile strength to 43.66 MPa with the strain at break unchanged at 480%. The strain at break is somewhat reduced relative to pure PP due to the rigid BT filler, but the interfacial adhesion provided by LSR107 mitigates embrittlement. This yields a composite that is both strong and ductile. The enhanced stress–strain behavior is consistent with the hierarchical interphase morphology and dense cross‐sectional microstructure evidenced by Figure 1, Figures S1, S8, and S15, and confirms effective polymer–interphase–filler integration. This synergistic enhancement arises from complementary roles, where LSR107 improves toughness by mitigating PP's inherent brittleness (with only a marginal strength sacrifice), and low BT loading (0.6–1.0 wt.%) counteracts this effect via stress transfer and matrix reinforcement. Beyond 1.2 wt.%, BT aggregation likely induces interfacial defects, reducing strength enhancement (Figure S18). Together, these results show that the ternary composite has sufficient mechanical robustness for flexible dielectric films or capacitors, satisfying industrial handling requirements.

Film capacitors are produced by roll‐to‐roll drawing to sub‐micrometer thickness, so high elongation at break is a fundamental processing requirement. A brittle film will fail during such drawing, regardless of its intrinsic dielectric performance. To assess whether the ternary composite can accommodate these stresses, finite‐element simulations were performed (Figure 4b). Two idealized 2D unit cells were modeled: one of PP with 1 wt.% BT (top row of Figure 4b) and one with an additional compliant LSR107 interphase surrounding an idealized BT inclusion (bottom row). The undeformed unit cells are shown on the left, and the right panels plot von Mises stress contours under 50% and 100% uniaxial stretch. In the PP/BT model, the rigid BT inclusions generate high stress concentrations in the narrow polymer ligaments. By contrast, the PP/LSR107/BT model (with the compliant LSR107 shell) shows that these local stress peaks are greatly reduced, yielding a much more uniform stress distribution at high strain. This interphase‐buffered architecture thus mitigates localized strain amplification that would otherwise initiate cracks during extreme stretching.

The thermal behavior of the PP/LSR107/BT ternary composite (Figure 4c,d and Figure S17) remains close to that of neat PP. The PP melting temperature shows no pronounced change and stays near 165°C across the studied compositions, indicating that LSR107 and BT have a minimal impact on the melting transition. By contrast, the crystallization temperature (T_c_) is more sensitive to composition. Increasing LSR107 shifts T_c_ to lower values, whereas introducing BT raises Tc markedly and can even bring it slightly above that of neat PP, consistent with BT providing effective heterogeneous nucleation while preserving the overall thermal profile of PP. Importantly, the melting point remains well above 150°C, supporting thermal reliability under industrial operating conditions. At BT loadings of 1 wt.% or higher, β‐phase‐related structural changes become more evident in the XRD/thermal characterization (Figures S4 and S17), which correlates with enhanced tensile ductility. This β‐phase formation is not observed in neat PP or the PP/LSR107 binary blend and enables the optimal ternary composite to retain over 480% strain (Figure 4a).

Figure 4e compares the electrical energy storage of the various composite dielectrics. The ternary PP/LSR107/BT composites markedly outperform both unfilled PP and the PP/LSR107 binary at the breakdown threshold, the PP/4 wt.% LSR107/1 wt.% BT film achieves a discharged energy density of 5.89 J cm^−^^3^ at 96% efficiency, whereas neat PP reaches only 2.53 J cm^−^^3^, and PP/4 wt.% LSR107 about 3.93 J cm^−^^3^. This performance boost arises from complementary contributions. LSR107 enhances breakdown strength and suppresses dielectric loss, whereas BT increases permittivity (Figure S13), and their combination enables substantially higher energy storage. In all cases, the films maintain over 90% efficiency even at high electric fields. These results highlight that the ternary‐component blend unlocks a synergistic energy storage advantage that neither component alone can provide.

Figure 4f demonstrates excellent cycling stability of the best‐performing film (PP/4.0 wt.% LSR107/1 wt.% BT) under realistic conditions (500 kV mm^−^^1^ pulses at 100 Hz). After 10^5^ repeated charge‐discharge cycles, the discharged energy density remains essentially constant, and the efficiency stays above 93%. No dielectric breakdown or fatigue is observed. Such high cycle endurance, retaining nearly full energy density and efficiency after 1 00 000 cycles, confirms the material's long‐term reliability for capacitor applications.

Critical for industrial translation, the PP/LSR107/BT platform is benchmarked against representative dielectric optimization strategies to visualize the coupled requirements of high energy density, high efficiency, and manufacturable mechanics (Figure 4g) [16, 54, 55, 56, 57, 58, 59, 60, 61, 62, 63, 64, 65, 66, 67, 68]. Bubble size is proportional to elongation at break, capturing the strain tolerance needed for continuous melt extrusion and high ratio drawing without tearing or void formation. This metric is a processing gate rather than an auxiliary property, because industrial thinning to sub‐tens of micrometres inherently imposes extreme tensile deformation on the melt‐drawn film. If the dielectric is embrittled by fillers or interfaces, mechanical failure and defect generation become the dominant limitation, undermining thickness uniformity and reliability even when the intrinsic dielectric metrics appear promising. To benchmark application‐relevant performance, emphasis is placed on data points that sustain electric fields above 600 kV/mm while maintaining charge–discharge efficiency above 90%. This criterion targets the upper‐end field endurance typically associated with capacitor‐grade BOPP and screens out formulations whose conduction loss rises sharply at high fields, which would otherwise erode efficiency under practical operating conditions. Achieving a discharged energy density above 5.5 J cm^−^^3^ under these constraints therefore represents a meaningful step beyond typical polypropylene film performance and directly translates into capacitor miniaturization at practical efficiency. Consistent with this criterion, the ternary composite uniquely combines a discharged energy density exceeding 5.5 J cm^−^^3^ at over 90% efficiency together with retained ductility above 480%, indicating that electrical gains are achieved without sacrificing the stretchability needed for roll‐to‐roll manufacturing.

## 3 Conclusion

In summary, this work has developed a flexible hierarchical PP/LSR107/BT ternary‐component composite dielectric film that overcomes the intrinsic permittivity–breakdown trade‐off of conventional PP films. A scalable one‐step extrusion‐and‐stretching process yields meter‐scale uniform films with LSR107 confined to PP's amorphous regions, forming compliant LSR107‐rich interphases around local BT clusters / BT‐rich regions. This architecture, combined with the deep trap states introduced by LSR107, homogenizes the electric field and suppresses leakage. As a result, the composite simultaneously achieves high permittivity and ultrahigh breakdown strength, delivering a record discharge energy density of 5.89 J cm^−^^3^ at 96% efficiency. It also shows greatly enhanced mechanical robustness (over 67% higher tensile strength at 43.7 MPa) and ductility (above 480%), with a void‐free morphology, ensuring industrial reliability. Benchmarking confirms this system outperforms existing PP‐based dielectrics across key metrics. This approach, merging molecular‐level confinement with interfacial band engineering, establishes a general design paradigm for scalable high‐performance polymer dielectric films applied in electrostatic capacitors.

## 4 Experimental Section

### 4.1 Materials

All chemicals were used as received without further purification. Polyvinylpyrrolidone (PVP, *M_w_* ≈ 58,000), barium hydroxide octahydrate (Ba(OH)_2_·8H_2_O, 99.95%), tetrabutyl titanate (Ti(OC_4_H_9_)_4_, 99%), and triethylene glycol (TEG, ≥ 99%) were purchased from Aladdin (China). Xylene (≥ 98.5%), isopropanol (≥ 99.7%), and vinyl‐terminated polydimethylsiloxane (LSR107 5000cst) were obtained from Macklin. Nile Red (analytical grade) was acquired from Shanghai Acmec Biotech, and isotactic polypropylene (PP, J802) was supplied by Lanzhou Petrochemical. The rest of the materials, if not mentioned specifically, were supplied by Aladdin, China.

### 4.2 Characterizations

Structural and morphological analysis was performed using field‐emission scanning electron microscopy (FE‐SEM, JEOL JSM‐6301F, Japan) to examine film cross‐sections and elemental distribution. Transmission electron microscopy (TEM, Tecnai Spirit, FEI, USA) characterized the size and elemental composition of nanoscale BaTiO_3_ (BT) particles. Atomic force microscopy (AFM, Dimension Icon, Bruker, Germany) assessed surface nanomechanical properties of the composites. Film morphology after staining was imaged using Polarized optical microscopy (POM, DM4 M, Leica, Germany) coupled with a confocal laser scanning microscope (CLSM, FV3000, Olympus, Japan). Film composition, crystallinity, and thermal properties were investigated using X‐ray diffraction (XRD, D/max‐2550, Rigaku, Japan) with Cu Kα radiation, Fourier‐transform infrared spectroscopy (FT‐IR, TG209 Libra, NETZSCH, Germany), and Differential Scanning Calorimetry (DSC, DSC8000, PerkinElmer, USA). Fracture elongation was determined by tensile testing (3343 Load Frame, Instron, USA). Dielectric properties were measured across a frequency range of 10^−^^1^ to 10^7^ Hz using broadband dielectric spectroscopy (Concept 40, Novocontrol Technologies, Germany). Dielectric breakdown strength was determined using a dielectric withstand testing system (HJCK‐50kV, Beiguang Jingyi, China). Unipolar electric displacement loops (D‐E) were measured at 100 Hz using a ferroelectric tester (Premier II, Radiant Technologies, USA). Leakage current characteristics were evaluated using a high‐voltage source (2290‐10, Keithley, USA) combined with a source measure unit (2635B, Keithley, USA). Optical absorption spectra of neat PP and neat LSR107 were recorded by UV–vis spectroscopy (Lambda 950, PerkinElmer, USA) for bandgap estimation via Tauc plot analysis.

### 4.3 Synthesis of Barium Titanate (BT) Nanoparticles

BT nanoparticles were synthesized via a modified solvothermal route. Ba(OH)_2_·8H_2_O (5.68 g, 18 mmol) and PVP (0.75 g, 5 wt.% of theoretical BT yield) were dissolved in 50 mL of a 4:1 (v/v) TEG/deionized water mixture under stirring (500 rpm, 25°C, 30 min). Ti(OC_4_H_9_)_4_ (5.1 mL, 15 mmol) was added dropwise to Ba: Ti = 1.2:1. The precursor was sealed in a 100 mL Teflon‐lined autoclave and heated at 160°C for 12 h. After cooling, the product was washed with anhydrous ethanol (3 times) and 0.1 M formic acid (2 times), then vacuum‐dried at 80°C for 6 h. This yielded monodisperse perovskite BT (5 ± 2 nm) without high‐temperature calcination.

### 4.4 Fabrication of PP‐Based Dielectric Films

Composite films were produced via an integrated mixing‐stretching process. Before melt blending, the BT nanoparticles were ultrasonically dispersed in ethylene glycol, dried, and subjected to a further deagglomeration‐assisted treatment. Pre‐blending PP pellets, LSR107 elastomer (4 wt.%), and BT nanoparticles (1 wt.%) were dry‐mixed in a planetary mixer (Thinky ARE‐310) using sequential low‐speed cycles (0.5 min, 800 rpm) and high‐speed cycles (5 min, 2000 rpm). The mixture was fed into a co‐rotating twin‐screw extruder (Thermo Scientific HAAKE PolyLab OS, L/D = 40) for melt compounding with defined temperature zones. Feed zone maintained at 180°C, compression zone at 200°C, homogenization zone at 210°C, and melt zone at 220°C. Screw speed was fixed at 100 rpm with 10 min residence time. The extruded melt was quenched to 50°C using an air knife (∆*T* ≈ 170°C/s), then uniaxially stretched at 500 mm/min with a draw ratio of 2.5× via differential speed rollers (Bruckner Karo IV lab stretcher) to film formation. Final bubble‐free composite films were wound at 40–60 N·m tension, achieving precise dimensions of 10 ± 0.2 µm thickness and 10 cm width (Figure 1c).

### 4.5 Nile Red Staining for Phase Visualization

Samples (3 × 3 cm) were pre‐swollen in xylene (25°C, 1 h) to promote diffusion into amorphous regions. They were then stained in 0.03 wt.% Nile Red/xylene (25°C, dark, 1 h). Because xylene preferentially swells LSR107, the dye selectively labels silicone‐rich domains. After ultrasonic washing in isopropanol (3 × 10 min, 40 kHz) and vacuum drying at 40°C for 24 h, films were imaged by CLSM (561 nm excitation) and POM.

### 4.6 Using the IRI Method, Calculate the Weak Intermolecular Forces

Intermolecular weak interactions between PP and LSR107 were analyzed by the Interaction Region Indicator (IRI). IRI is simply defined as follows

(2) $$\mathrm{IRI}\left(r\right)=\frac{|\nabla \rho \left(r\right)|}{{\left[\rho \left(r\right)\right]}^{a}},a=1.1$$

where is ρ(*r*) the electron density at coordinate*r*. Wavefunctions were computed, isosurfaces generated in Multiwfn (v3.8), and visualized in VMD (v1.9.3).

### 4.7 Density Functional Theory (DFT) Calculation of the Energy Levels

Frontier orbital energies of PP and LSR107 were computed at the ωB97X‐D level (Gaussian 16). After geometry optimization, single‐point energies yielded HOMO/LUMO. The gap was calculated as

(3) $$\Delta E=E_{\mathrm{LUMO}}-E_{\mathrm{HOMO}}$$

represent the respective orbital energies in electron volts (eV) to estimate the charge‐injection barrier and electronic insulation.

### 4.8 Finite‐Element Simulation of Local Electric Field

Local electric‐field distributions were simulated in a 2D representative volume element (RVE, 500 nm × 500 nm) containing idealized BT‐rich inclusions dispersed in PP, either without an interphase (PP/BT) or surrounded by an LSR107 interphase (PP/LSR107/BT). Linear isotropic dielectrics were assumed, with ε_*r*_(PP) = 2.3, ε_*r*_(LSR107) = 2.0, ε_*r*_(BT) = 1916. The BT permittivity was taken from the experimentally measured dielectric constant of a pressed‐and‐sintered BT pellet at ∼100 Hz (measured at 25°C after 1100°C sintering (Figure S10); the constitutive law was

(4) $$D=\varepsilon_{0}\varepsilon_{r}E$$

An average field of 550 kV mm^−1^ was applied across the thickness. The LSR107 shell reduces interfacial field concentration relative to PP/BT.

### 4.9 Finite‐Element Mechanical Simulation

Finite‐element simulations were performed in COMSOL Multiphysics 6.2 (Structural Mechanics Module) using the Solid Mechanics interface. A 2D unit cell (50 nm × 50 nm) contained four BaTiO_3_ inclusions (diameter 5 nm). For PP/LSR107/BT, an irregular LSR107 interphase was generated by creating a rough outer polygon around each BT particle and subtracting the BT circle. Geometries were generated via LiveLink for MATLAB and finalized by a Union operation while keeping interior boundaries, so each phase remained a distinct domain for material assignment. Each phase was modeled as isotropic linear elastic, with Young's modulus E and Poisson's ratio ν defined in the material database and referenced by the Linear Elastic Material feature. For the PDMS‐like LSR107 interphase, ν was set to 0.49 (rather than 0.5) to avoid incompressibility‐related numerical issues in linear elasticity. Uniaxial stretching was applied by prescribing the right‐edge displacement to achieve stretch ratios λ = 1.5 and 2.0, constraining the left edge in x, and fixing one point to remove rigid‐body motion. Geometric nonlinearity was enabled, and the mesh was locally refined at PP/BT and PP/LSR107 interfaces. Stress maps were post‐processed in the deformed configuration using the von Mises equivalent stress.

## Author Contributions

Z.M.D., K.B., and Y.G. conceived the idea; Y.G., K.B., Z.M.D., and S.L.Z. designed the experiments; Y.G. carried out most of the experiment work; K.H., X.J.W., L.H., K.X., J.T.W., Y.H.S., T.L., Z.P.F., and Y.M. helped with the high‐field dielectric measurements; Y.G., Z.M.D., K.B., S.L.Z., B.Q.W., L.J.Y. and L.H. analyzed the data. Y.G., Z.M.D., K.B. and S.L.Z. wrote the manuscript, and all authors participated in manuscript revision.

## Funding

Smart Grid‐National Science and Technology Major Project grant 2025ZD0808403 (ZMD); National Natural Science Foundation of China grant 52527803 (ZMD); National Natural Science Foundation of China grant 52437002 (ZMD); Zhongyuan Electric Laboratory Research Program grant ZC20241106 (ZMD); National Natural Science Foundation of China grant U2241243 (KB); Beijing Natural Science Foundation grant JQ22010 (KB).

## Conflicts of Interest

The authors declare no conflict of interest.

## Supporting information




**Supporting File**: advs75372‐sup‐0001‐SuppMat.docx.

## Data Availability

The data that support the findings of this study are available from the corresponding author upon reasonable request.
