# Supplementary material for: Flexible Unusual Ternary‐Component Graded‐Modulus Dielectric Films with High‐Density Capacitive Energy Storage
Source: Adv Sci (Weinh). 2026 Apr 24;13(40):e75372. doi: 10.1002/advs.75372 (PMC13335553; doi:10.1002/advs.75372)
Supplement: Supplementary file 1 — Supporting File: advs75372‐sup‐0001‐SuppMat.docx. [file ADVS-13-e75372-s001.docx]

Supporting Information

Flexible unusual ternary-component graded-modulus dielectric films with high-density capacitive energy storage

Yi Gao,^1,2,3^ Xin-Jie Wang,^2^ Lei Huang,^2,3^ Kun Xing,^1^ Baoquan Wan,^2^ Yan-Hui Song,^2^ Jian-Tao Wang,^2^ Li-Juan Yin,^2^ Tong Liang,^1^ Yan Meng,^1^ Zunpeng Feng,^1^ Kai Huang,^1^ Ke Bi,^1*^ Shao-Long Zhong,^2*^ Zhi-Min Dang,^2,3*^


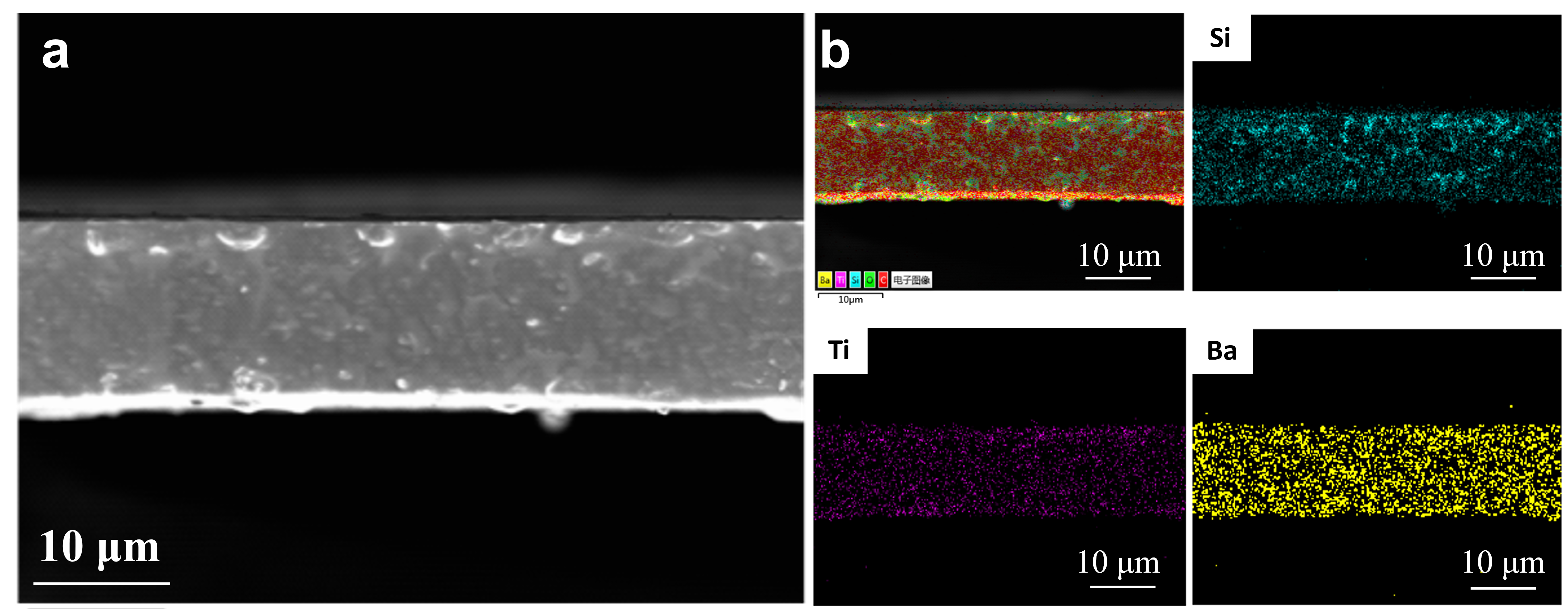


**Figure S1.** Cross-sectional morphology of PP/4.0 wt% LSR107/1.0 wt% BT composite film, a) SEM image, b) EDS elemental mapping. The ternary composite exhibits an overall homogeneous mesoscale distribution of LSR107- and BT-related regions across the film thickness, while nanoscale characterization reveals local BT clustering buffered by LSR107-rich interphases. This result supports the coexistence of mesoscale uniformity and nanoscale interphase-regulated heterogeneity in the composite.


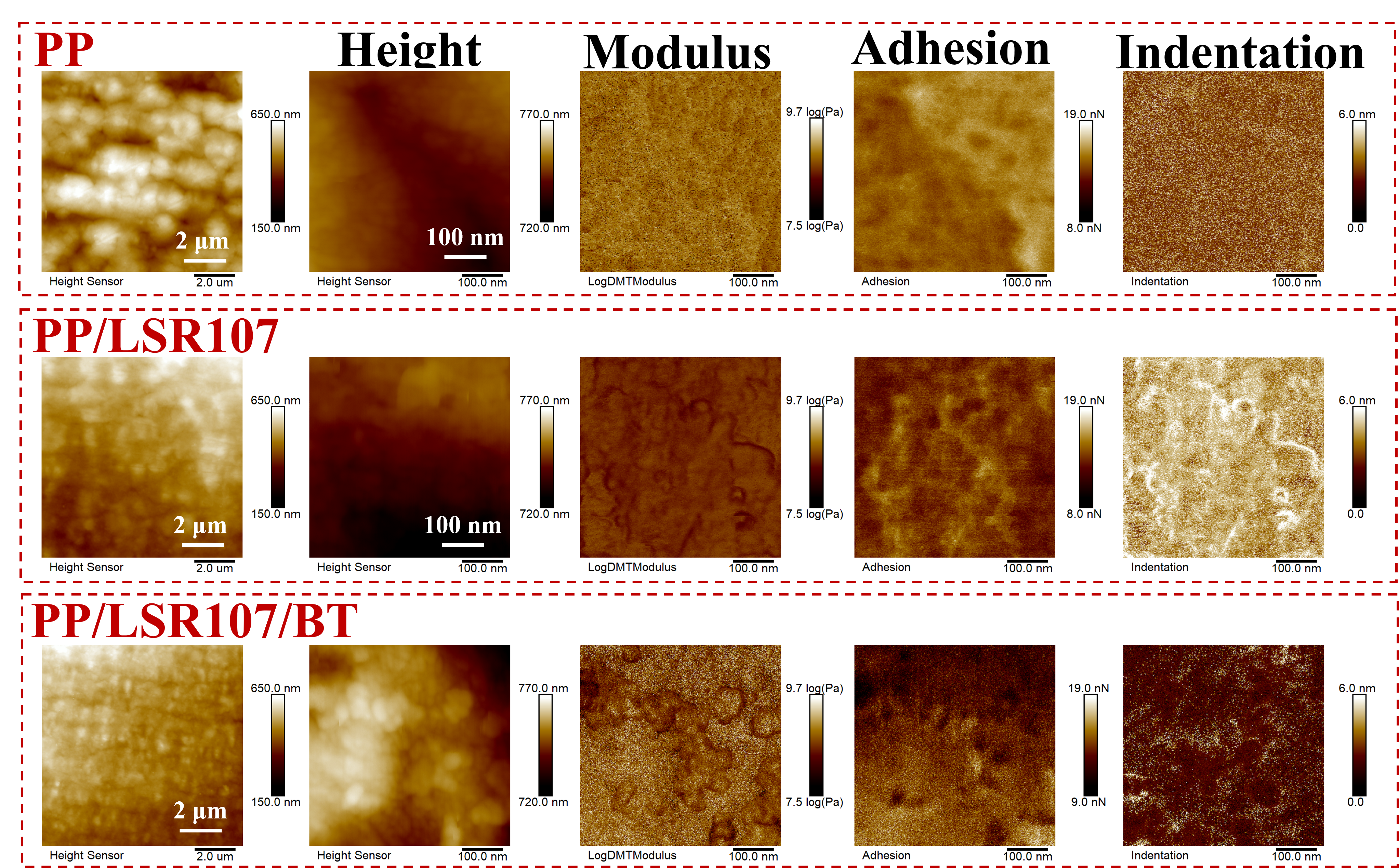


**Figure S2.** Quantitative nanomechanical AFM mapping along the stretching direction (Height, modulus, adhesion, and strain channels). Representative AFM phase images (top row) and corresponding quantitative nanomechanical maps (bottom row) are shown for PP, PP/4.0 wt% LSR107, and PP/4.0 wt% LSR107/1.0 wt% BT.


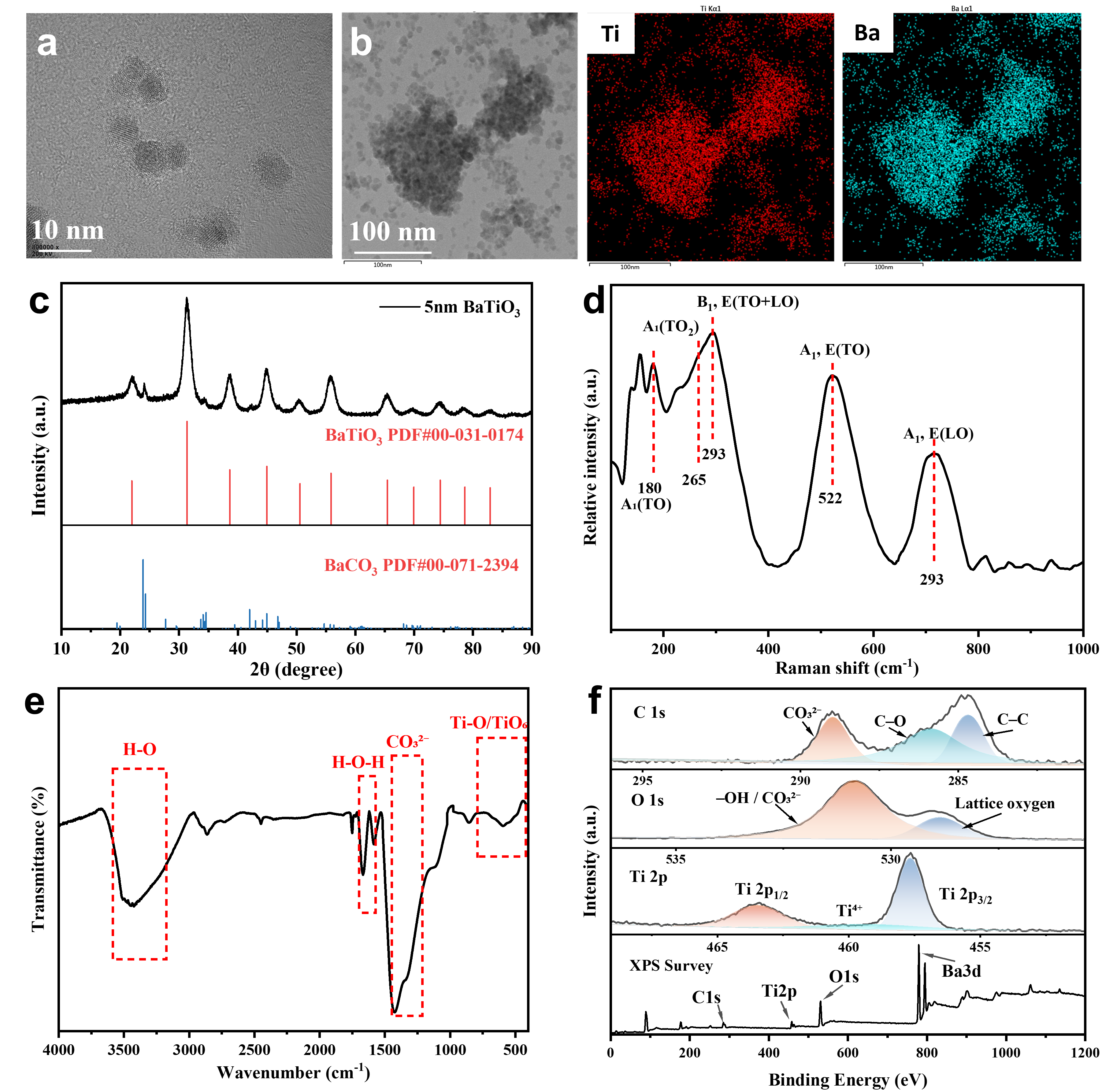


**Figure S3.** Structural characterization of BaTiO_3_ (BT) nanoparticles. a) TEM image of BT nanoparticles. b) TEM image and EDS elemental maps of Ti and Ba for BT aggregates, c) XRD pattern of BT nanoparticles together with reference PDF cards of BaTiO₃ and BaCO₃. The diffraction pattern is consistent with nanocrystalline perovskite BaTiO₃, while the absence of clearly resolved (002)/(200) splitting indicates that the XRD data do not support an unambiguous bulk-like tetragonal assignment. d) Raman spectrum of BT nanoparticles showing characteristic bands near ~254, ~295, ~512, and ~709 cm⁻¹, indicative of local tetragonal-like distortions / polar Ti off-centering. e) FT-IR spectrum of BT nanoparticles showing the BaTiO₃ lattice-vibration region together with minor O–H / H–O–H and carbonate-related absorptions, suggesting slight surface hydroxylation/carbonation. f) XPS survey and high-resolution spectra of Ti 2p, O 1s, and C 1s. The Ti 2p doublet is consistent with Ti⁴⁺ in BaTiO₃, while the O 1s and C 1s spectra reveal minor hydroxyl/carbonate-related surface species.

The BT nanoparticles display a narrow size distribution of about 5 nm, validating successful nanoparticle fabrication. EDS confirms a stoichiometric Ba/Ti/O composition, which is essential for maintaining high dielectric permittivity. The average crystallite size was estimated from the (110) reflection (2θ = 31.5°) using the Scherrer equation $D=K\lambda/(\beta cos\theta)$, where *K* = 0.9, *λ* = 0.15418 nm (Cu Kα), and *β* is the instrument-corrected full width at half maximum (in radians), yielding *D* = 5.4 nm, consistent with the TEM observation. Overall, BT nanoparticles are primarily nanocrystalline perovskite BaTiO₃. The XRD pattern alone does not justify a definitive bulk tetragonal assignment, whereas Raman spectroscopy indicates that local tetragonal-like distortions remain present. Meanwhile, FT-IR and XPS reveal minor carbonate/hydroxyl-derived surface species, which likely arise from surface adsorption/carbonation of the nanopowder rather than from a dominant bulk impurity phase.^[1-2]^


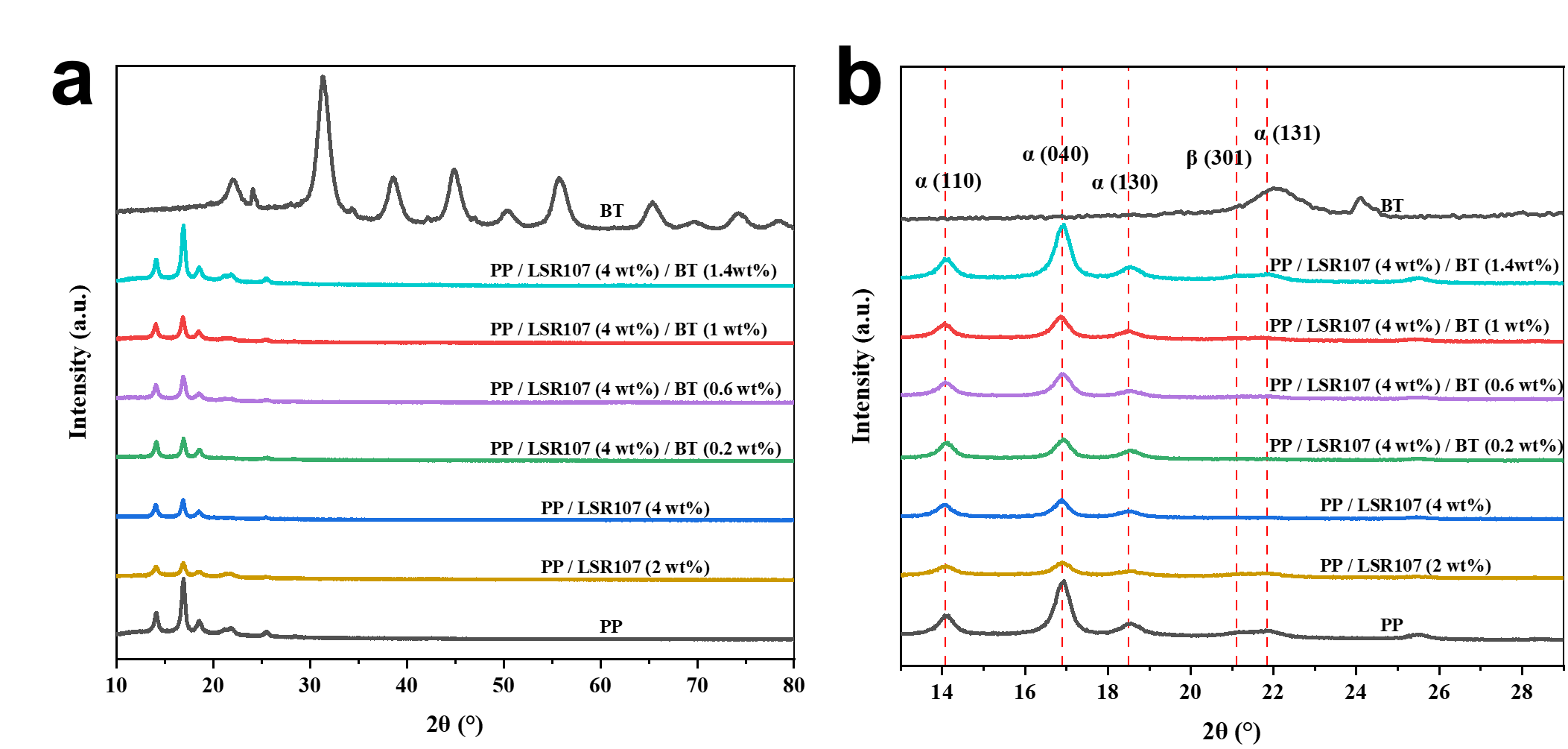


**Figure S4.** XRD patterns of polymer composites, a) PP/LSR107, b) PP/LSR107/BT. The incorporation of LSR107 reduces PP crystallinity by disrupting chain alignment, thereby improving toughness. The addition of BT increases the XRD peak intensities (a nucleation effect). No new crystalline phases or peak shifts are observed, confirming that only physical blending occurs (no new compound formation). PP remains predominantly in the α-phase (the β-phase peak at 2θ ≈ 21.1° is obscured by BT), consistent with the DSC results.


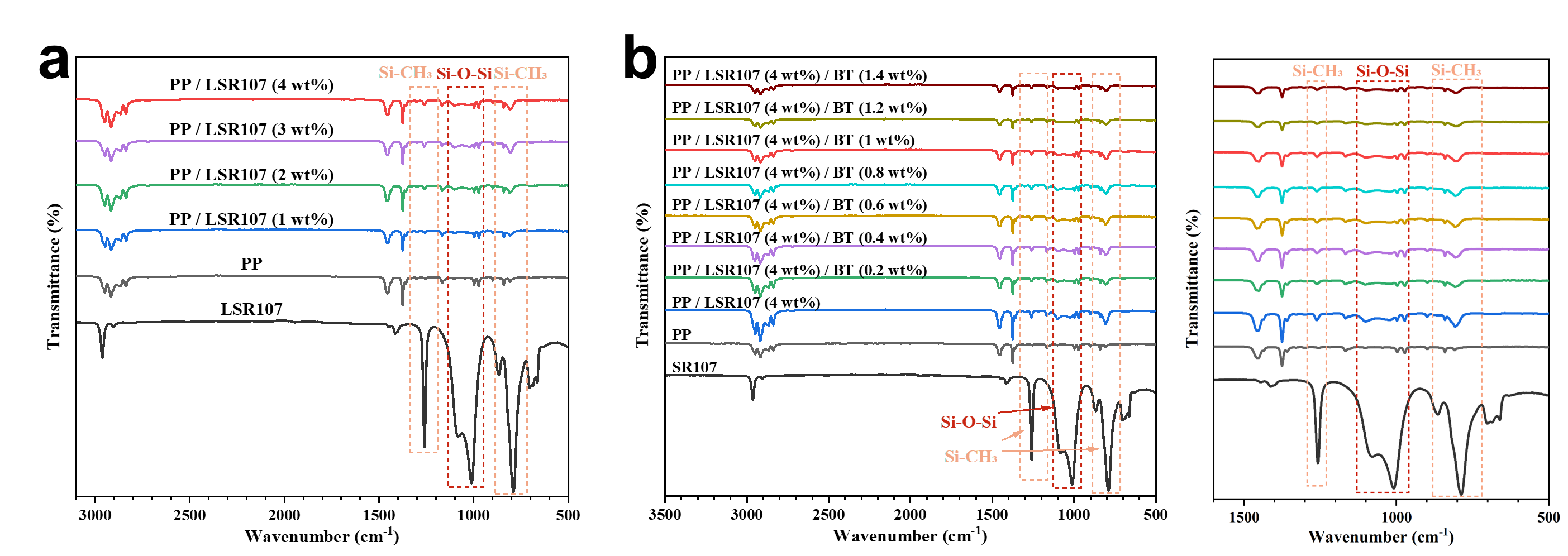


**Figure S5.** FT-IR spectra of composite films, a) PP with varying LSR107 content, b) PP/LSR107 (4 wt%) with varying BT content. LSR107 physically blends with PP, with no evidence of chemical bonding between the two. The introduction of BT produces two effects on the spectra: (i) Physical shielding – infrared scattering by the BT particles reduces spectral transmittance (dominant at higher BT loadings), (ii) Interfacial interactions – hydrogen bonding and dipolar interactions between BT and LSR107 broaden or shift the Si–O–Si absorption peaks, thereby enhancing interfacial polarization.


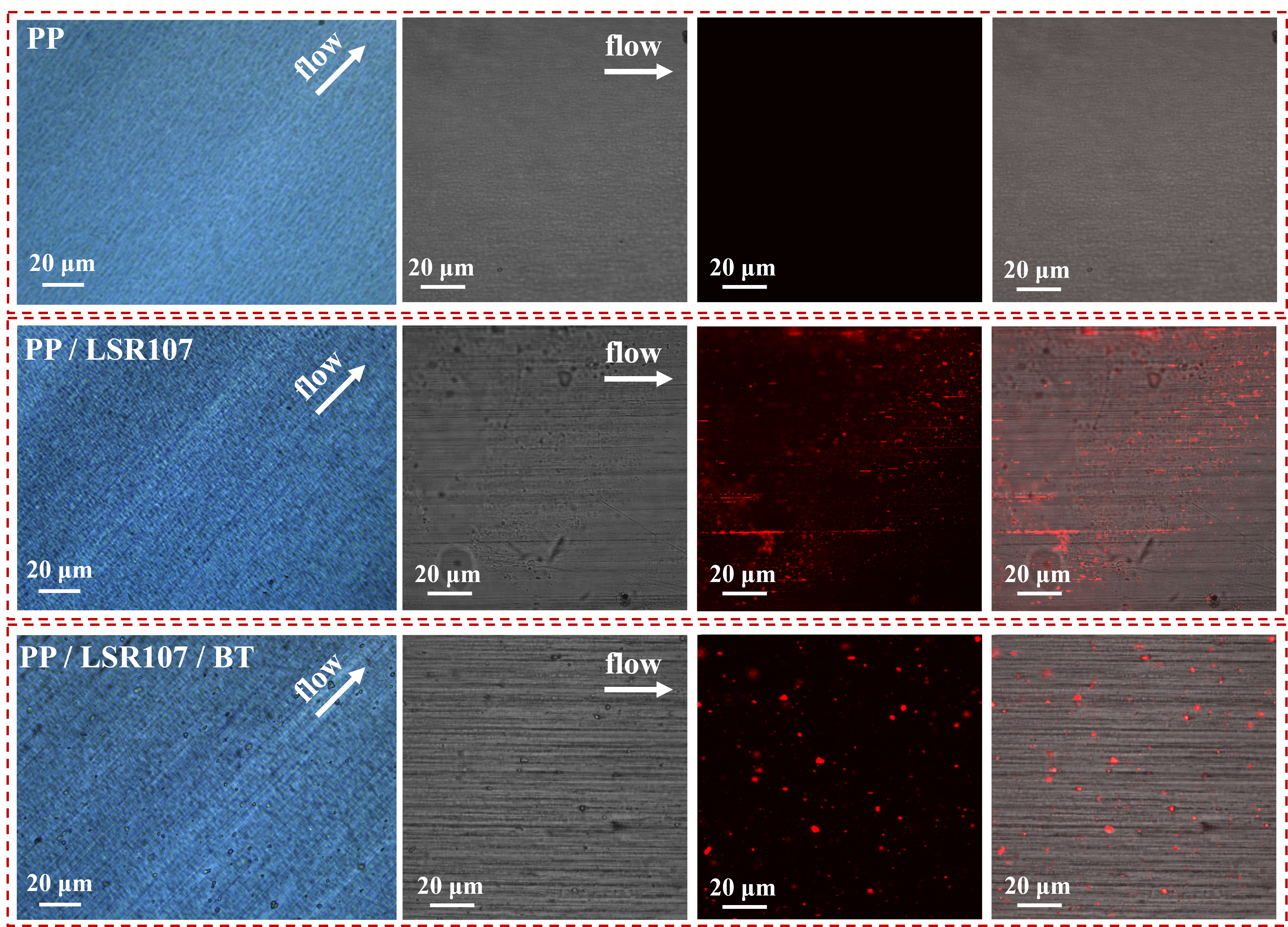


**Figure S6.** Solvent swelling with Nile Red staining under multimodal microscopy. Shown are polarized light microscopy (PLM) images (left column) and confocal laser scanning microscopy (CLSM) images (right columns: bright field, dark field, and DIC) for PP, PP/4.0 wt% LSR107, and PP/4.0 wt% LSR107/1.0 wt% BT. The selective Nile Red staining highlights the distribution of the LSR107 phase. Under polarized light, the drawn PP exhibits highly oriented “shish-kebab” crystalline structures, whereas the CLSM fluorescence images show the red-emitting LSR107-rich domains aligned along the stretching direction together with BT-related bright features associated with local BT-rich regions.


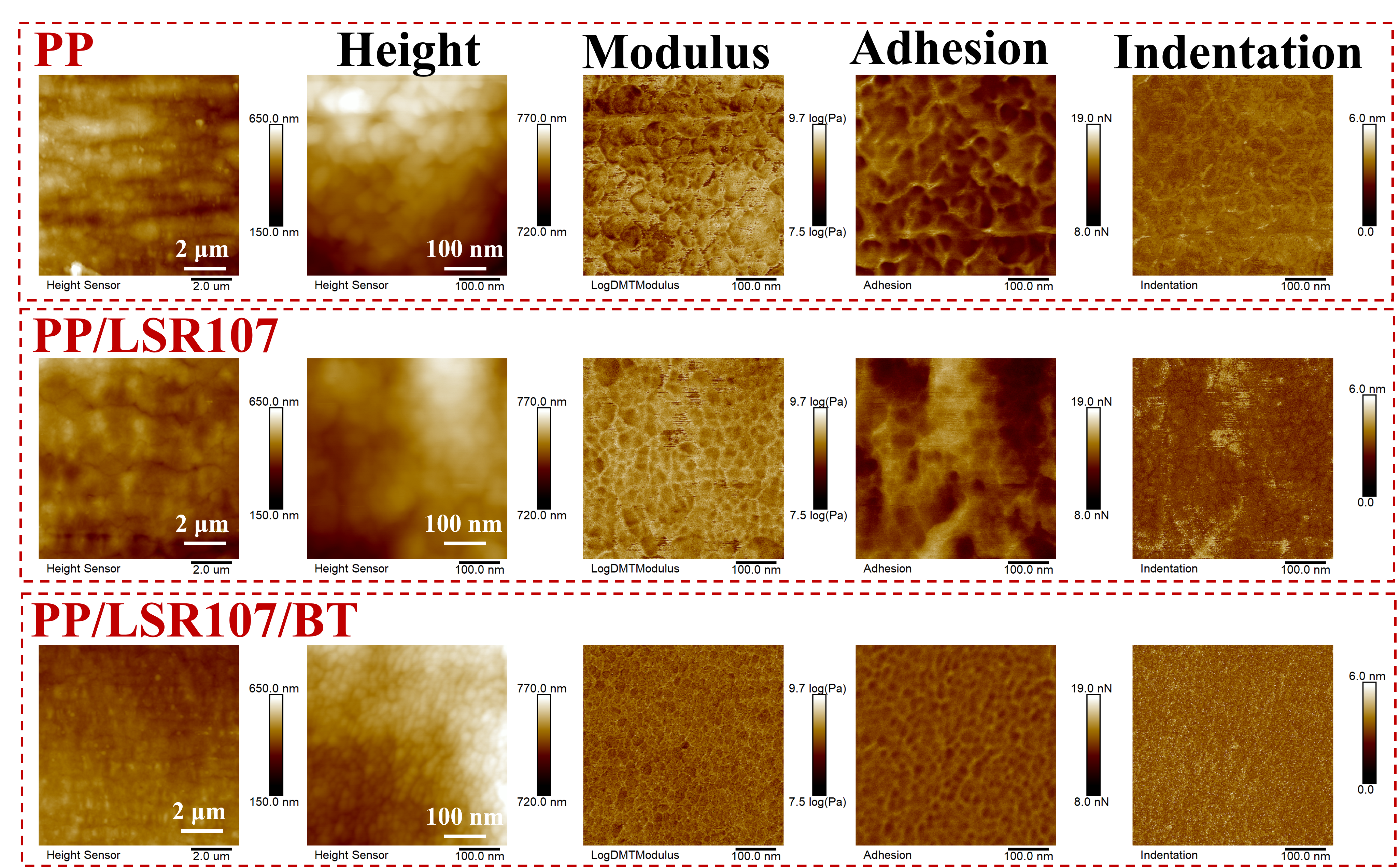


**Figure S7.** AFM morphology mapping of Nile Red-stained films. Top to bottom: PP, PP/4.0 wt% LSR107, PP/4.0 wt% LSR107/1.0 wt% BT, left to right: crystalline phase (aligned with stretch direction), height, modulus, viscosity, and strain maps. Xylene vapor swelling for 1 h preserves the polymer’s crystalline structure but induces nanoscale swelling of the amorphous regions. The BT-filled composites exhibit higher modulus and viscosity in the AFM maps, indicating reinforced nanoscale mechanical stability.

**
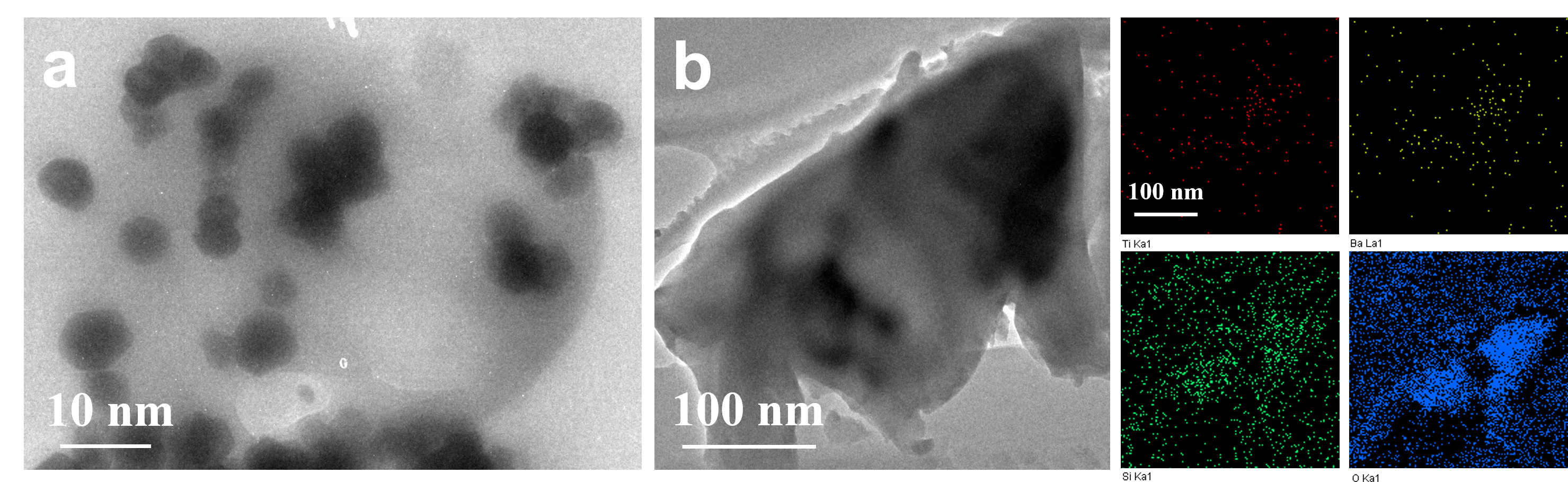
**

**Figure S8.** a) TEM image showing that the dark-contrast BT nanoparticles are not completely isolated as individual particles after melt processing, but remain locally clustered on the nanoscale. The surrounding lighter-contrast region indicates that these BT-rich regions are embedded in and broadly wrapped by LSR107-rich domains. b) TEM image at a larger scale together with the corresponding STEM-EDS elemental maps, showing a BT-rich region surrounded by a more spatially extended matrix domain. The elemental distributions indicate that the Ba/Ti-rich region is locally confined, whereas the Si/O-containing region extends more broadly around it. This results suggest that LSR107 forms a wider compliant interphase around BT-rich regions, corresponding to partial/broad encapsulation rather than a perfectly uniform particle-by-particle shell.

**
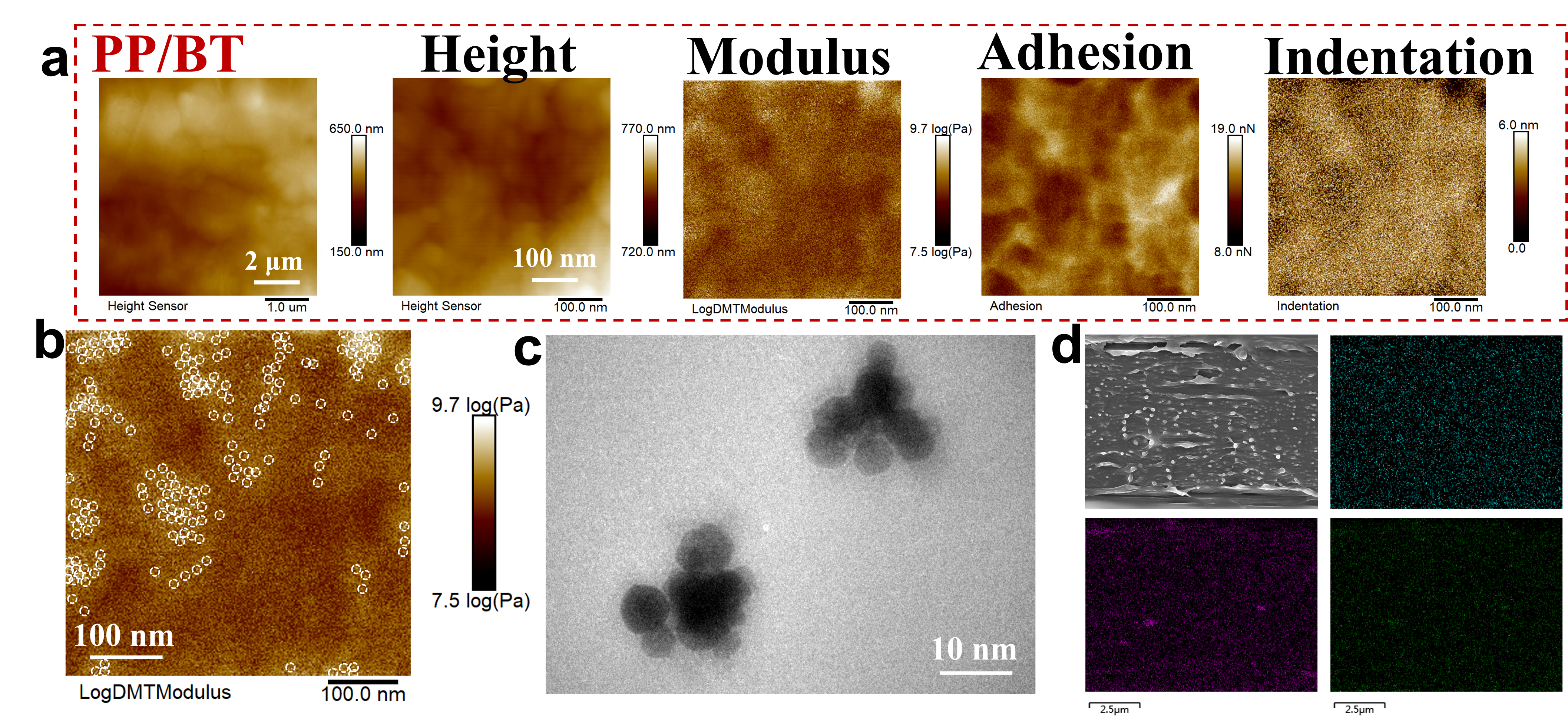
**

**Figure S9.** Microstructure characterization of the PP/BT (1 wt%) binary composite film. a) Quantitative nanomechanical AFM mapping images of the PP/BT (1 wt%) film. From left to right: crystalline phase (aligned with the stretch direction), height, modulus, viscosity, and strain maps. b) Enlarged modulus map, in which the BT-rich regions are highlighted by white dashed circles to visualize their spatial distribution. c) TEM image of the PP/BT (1 wt%) film. d) Cross-sectional SEM image and corresponding EDS elemental maps (Ba, O, and Ti) of the PP/BT (1 wt%) film.

In Figure S9a, the AFM maps show the crystalline phase aligned with the stretching direction together with the corresponding height, modulus, viscosity, and strain responses. The modulus-related contrast indicates the presence of locally rigid nanoscale features associated with BT incorporation. In the enlarged modulus map (Figure S9b), these BT-rich regions are highlighted by white dashed circles, showing that the filler is distributed as discrete local nanoscale features. The TEM image (Figure S9c) further confirms that BT remains locally aggregated/clustered in the PP matrix. The SEM/EDS results (Figure S9d) show that Ba- and Ti-containing features are overall uniformly distributed across the observed micrometer-scale region of the PP matrix, indicating the absence of obvious large-scale/macroscopic segregation, while the nanoscale observations still reveal local BT clustering.

**
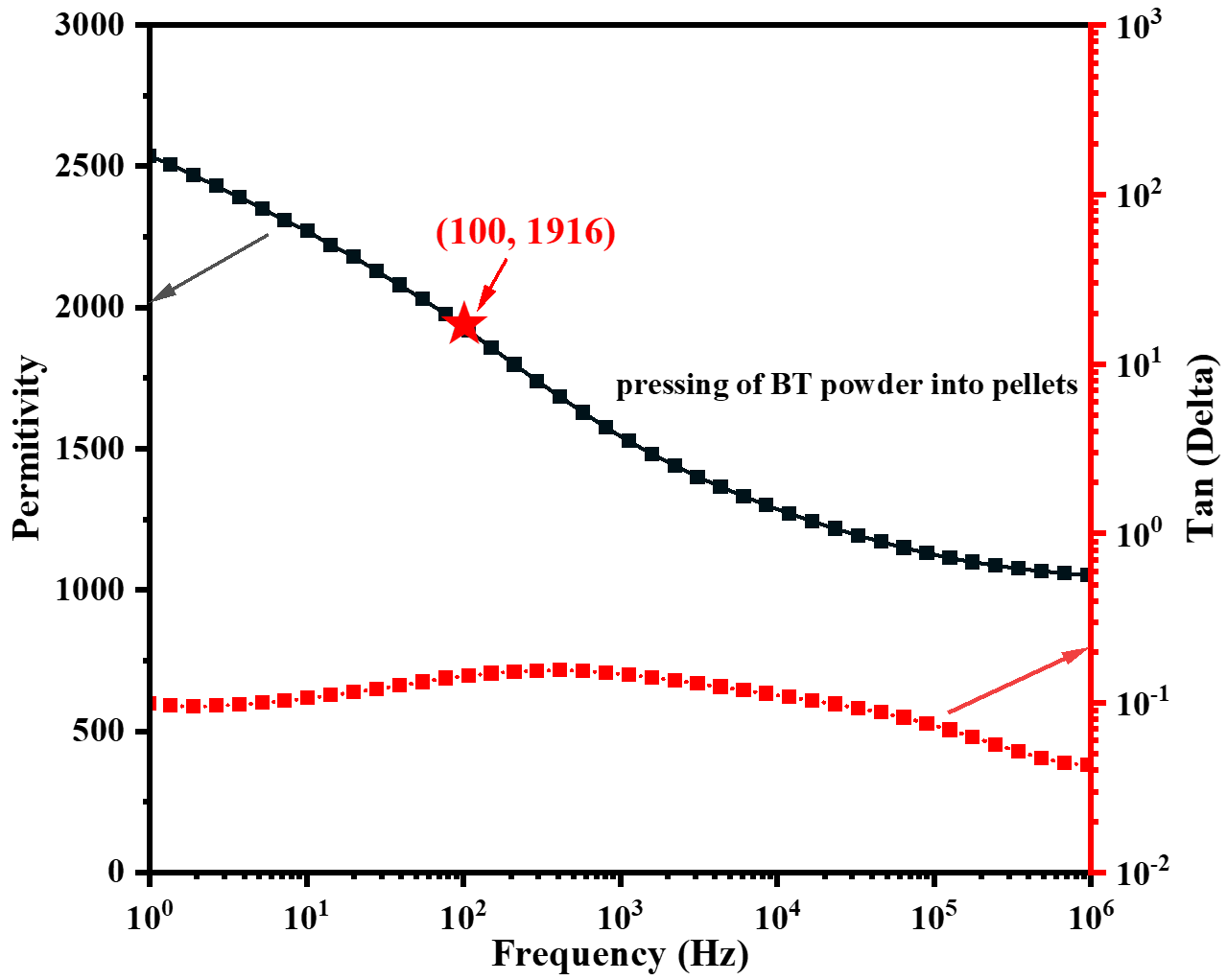
**

**Figure S10.** Frequency-dependent relative permittivity (εr) and dielectric loss (tan δ) of a BT pellet prepared by pressing the BT powder followed by sintering at 1100 °C. The dielectric measurement was carried out at room temperature (25 °C). The inset shows the corresponding BT pellet used for the dielectric test. The measured effective dielectric constant is 1916 at ~100 Hz, and this experimentally determined value was adopted as the dielectric constant of BT in the revised finite-element simulation.


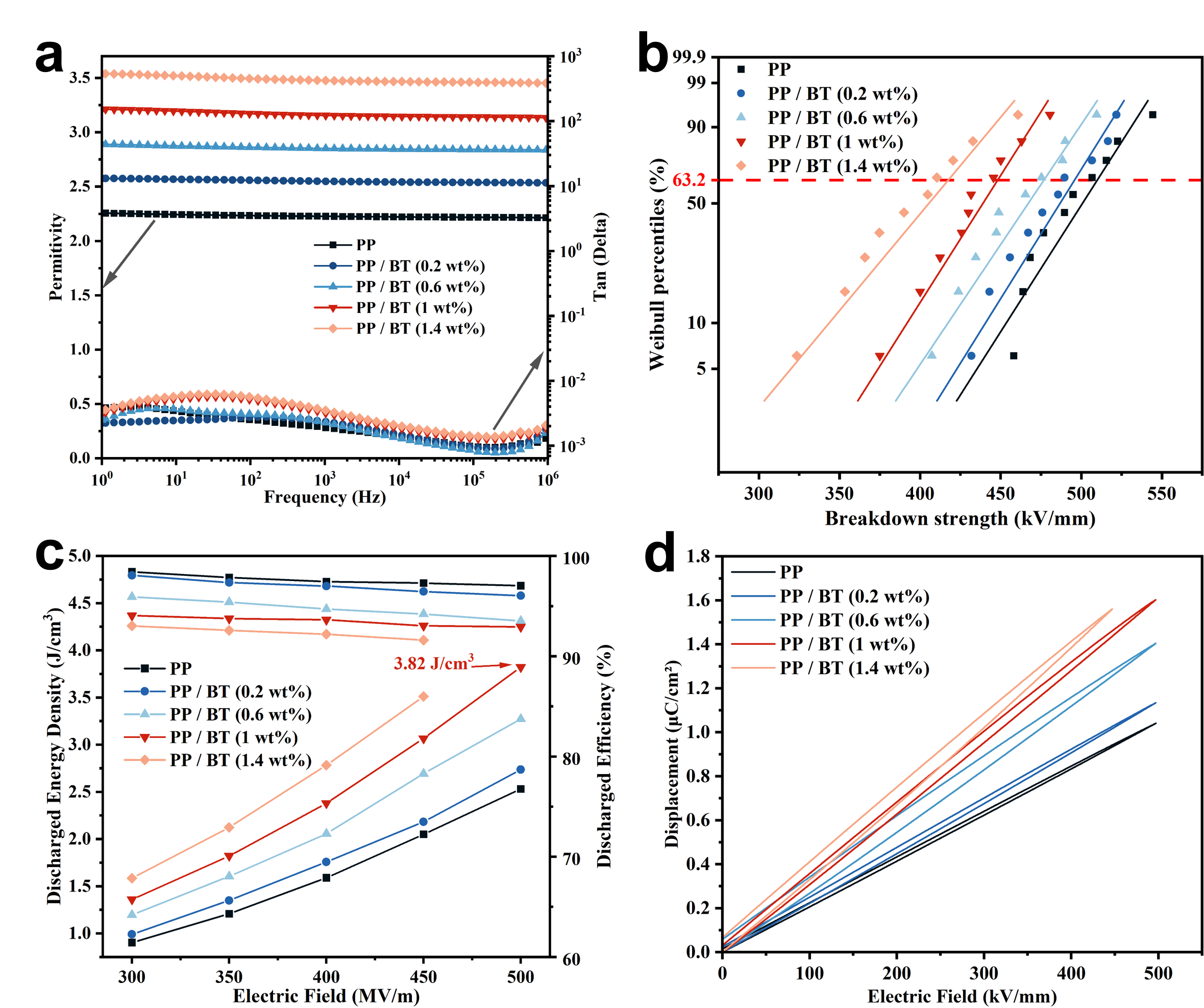


**Figure S11.** Dielectric performance of BT/PP composite films, a) Frequency-dependent ε_r_ and tan δ, b) Weibull breakdown strength, c) discharged energy density (U_e_) and efficiency, d) D–E loops. Adding BT increases the dielectric constant (ε_r_) of PP with only a slight rise in loss (tan δ). The optimal 5 nm BT content (≈1 wt%) yields the highest energy density U_e_ along with >90% discharge efficiency, with slim D–E loops confirming minimal hysteresis loss in the material.^[3]^


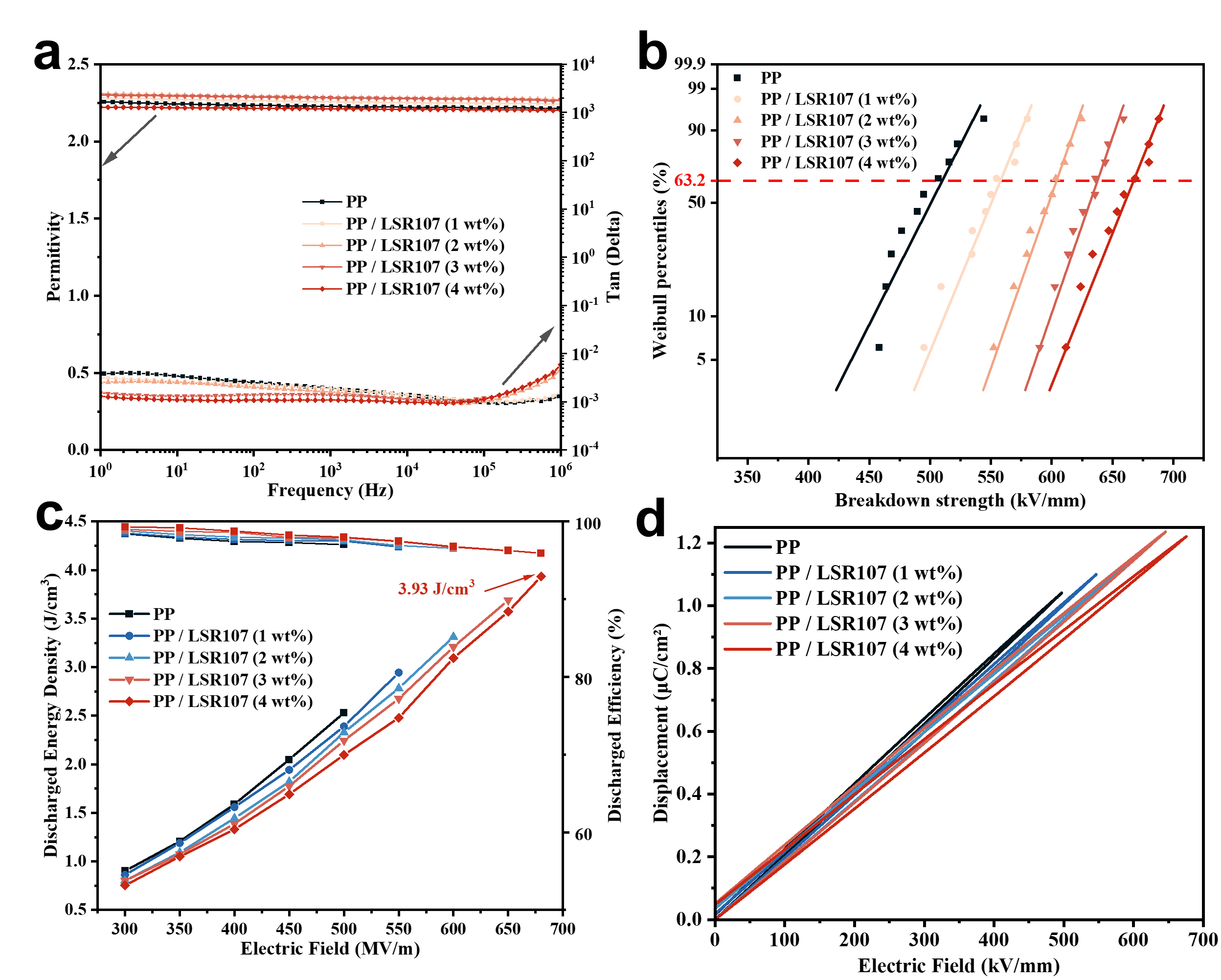


**Figure S12.** Dielectric performance of BT/LSR107 composite films, a) Frequency dependence of relative permittivity (εr) and dielectric loss (tan δ). b) Weibull distribution of breakdown strength (E_b_). c) Discharged energy density (U_e_) and efficiency as a function of electric field. d) D–E loops. Incorporating LSR107 (1–4 wt%) causes a slight decrease in ε_r_ while maintaining consistently low tan δ across the measured frequency range. In contrast, Eb is markedly enhanced with increasing LSR107 content, which dominates the energy-storage behavior and enables higher U_e_ at comparable efficiency.


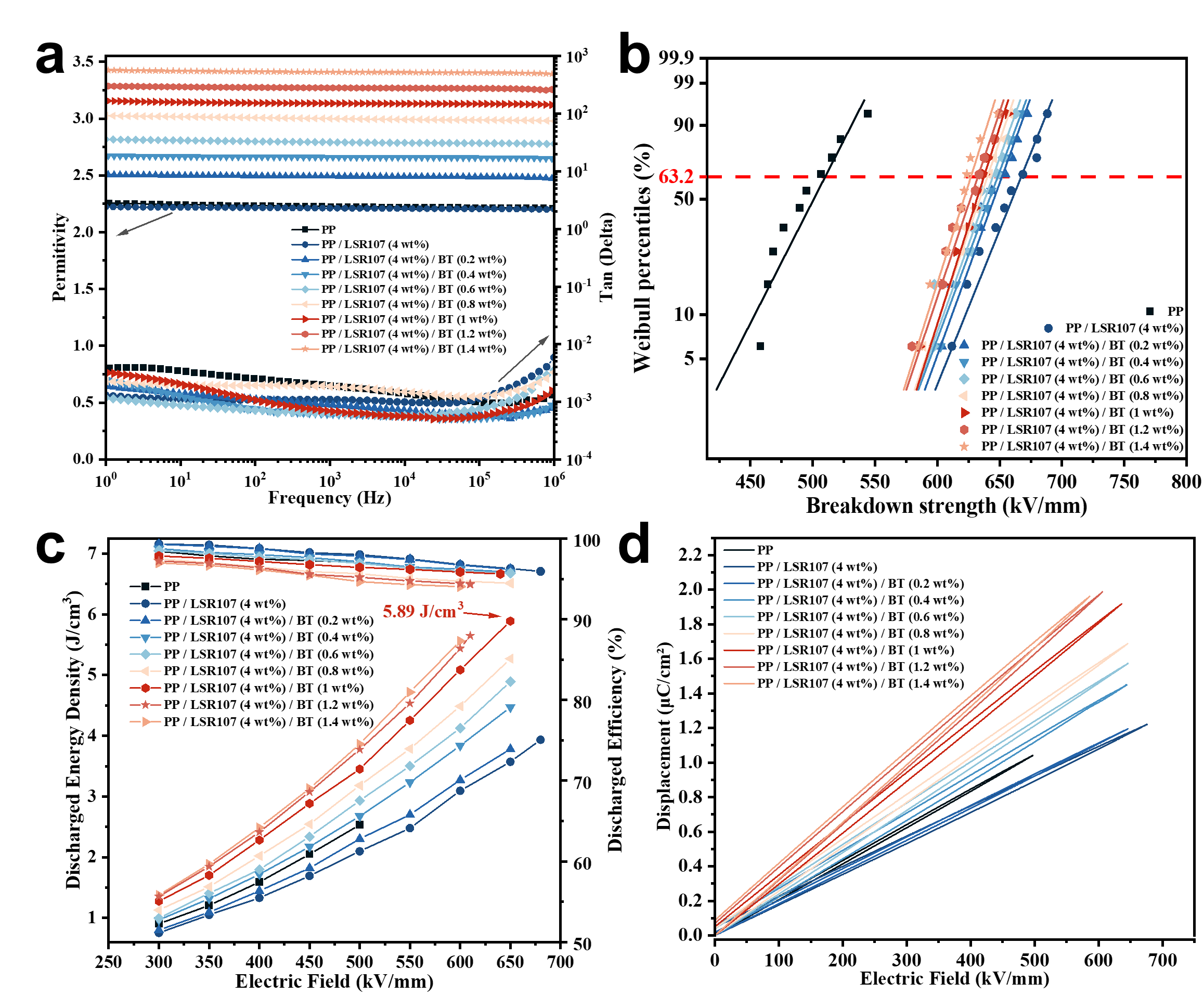


**Figure S13.** Dielectric performance of ternary BT/LSR107/PP composite films, a) Frequency-dependent ε_r_ and tan δ, b) Weibull breakdown strength, c) discharged energy density (U_e_) and efficiency, d) D–E loops. The ternary composite achieves a balanced improvement in permittivity and breakdown strength, leading to high energy density and efficiency with low dielectric loss.^[4]^


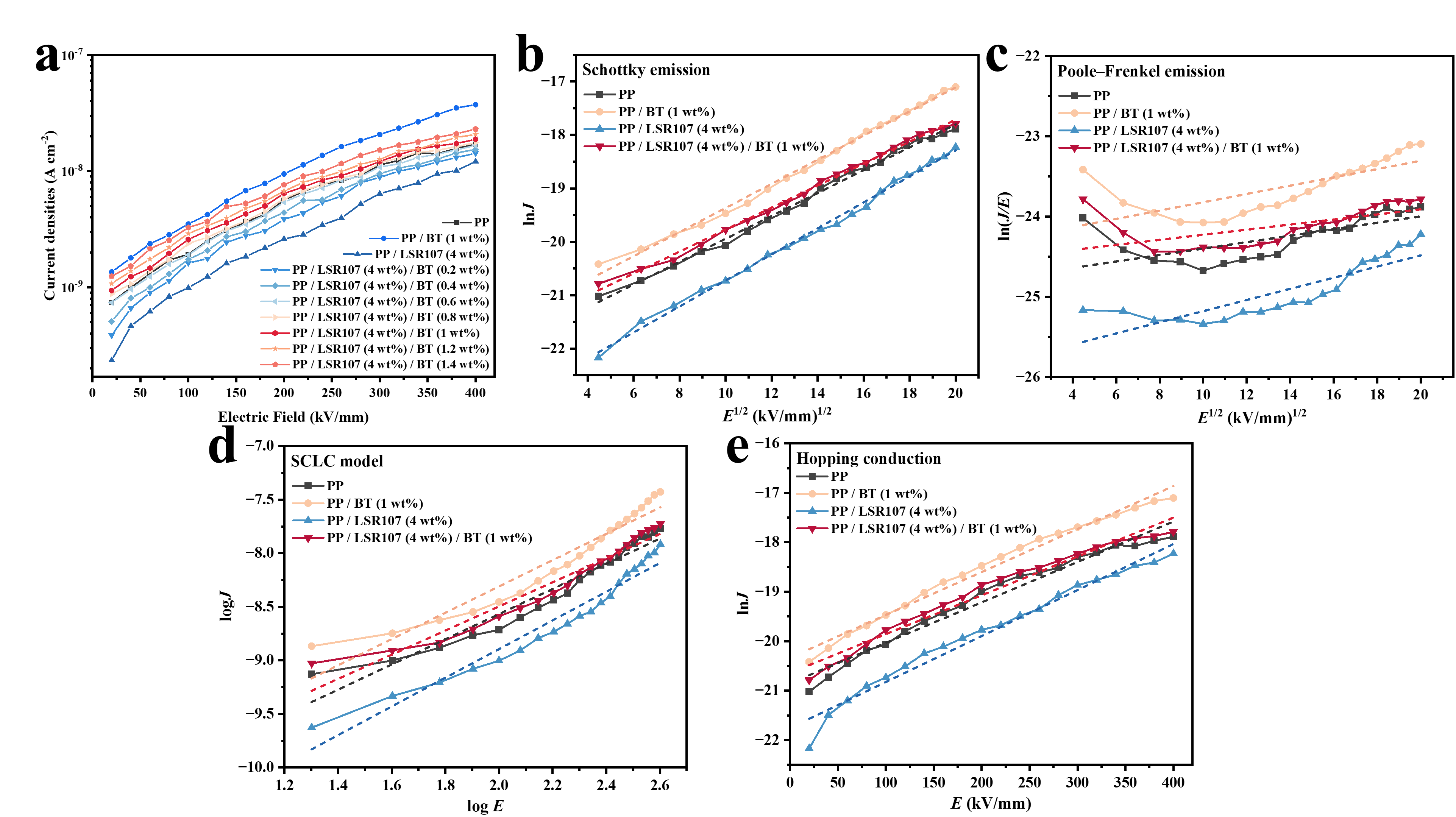


**Figure S14.** Leakage-current analysis of PP-based films and conduction-model fitting. **a)** Leakage current density as a function of electric field for PP-based films with varied BT and LSR107 contents. **b–e)** Conduction-model analyses of four representative films, namely neat PP, PP/BT (1 wt%), PP/LSR107 (4 wt%), and PP/LSR107/BT (1 wt%), based on **b)** Schottky emission, **c)** Poole–Frenkel emission, **d)** space-charge-limited conduction (SCLC), and **e)** hopping transport. Symbols represent experimental data and dashed lines represent fitted curves.

Leakage current density as a function of electric field for neat PP, PP/4.0 wt% LSR107, and PP/4.0 wt% LSR107 films containing different BT loadings (0.2–1.4 wt%). Incorporating 4.0 wt% LSR107 significantly suppresses the leakage current compared to neat PP, indicating improved insulating behavior under high fields. After introducing BT, the leakage current increases moderately, consistent with the enhanced carrier transport pathways associated with ceramic fillers. Notably, further increasing BT up to 1.0 wt% does not induce a disruptive rise in leakage current, suggesting that the composite maintains stable conduction loss within the investigated filler range.

To further elucidate the carrier-transport behavior in the PP-based films, the leakage-current data were analyzed using four commonly considered conduction models, namely Schottky emission, Poole–Frenkel emission, space-charge-limited conduction (SCLC), and hopping conduction (Figure S14b–e). According to the general classification of dielectric-film transport, Schottky emission is an electrode-limited mechanism, whereas Poole–Frenkel emission, hopping conduction, and SCLC are bulk-limited mechanisms^[5]^.

For Schottky emission, the current density is given by

where $A^{*}$is the effective Richardson constant, $q\Phi_{B}$is the Schottky barrier height, and $\varepsilon_{r}$is the optical dielectric constant. Accordingly, a standard Schottky plot should exhibit a linear relationship between $\log(J/T^{2})$and $E^{1/2}$.

For Poole–Frenkel emission, the field-assisted detrapping current can be expressed as

where $q\Phi_{T}$is the trap barrier height. In this case, the characteristic linearized representation is $\ln(J/E)$versus $E^{1/2}$. It should be noted that Poole–Frenkel emission is highly sensitive to trap concentration, and in the modified Poole–Frenkel regime its slope may approach that of Schottky emission, making the distinction between the two mechanisms nontrivial without additional temperature- or electrode-dependent measurements.

For SCLC, the current density generally follows

$$J\propto E^{n}$$

and, in the ideal trap-free limit, can be written as the Mott–Gurney relation

$$J=\frac{9}{8}\varepsilon_{r}\varepsilon_{0}\mu\frac{E^{2}}{d}$$

where $\mu$is the carrier mobility and $d$is the film thickness. Therefore, SCLC is usually analyzed using a $\log(J)$versus $\log(E)$plot, in which the slope $n$is used to identify ohmic, trap-filled-limited, and Child’s-law regions.

For hopping conduction, the current density may be described by

$$J=qan\nu\exp\left(\frac{qaE-E_{a}}{kT}\right)$$

where $a$is the mean hopping distance between trap sites, $n$is the carrier concentration, $\nu$is the thermal vibration frequency, and $E_{a}$is the activation energy from trap states to the conduction band edge. In this model, charge transport occurs by tunneling-assisted hopping between localized trap sites. Therefore, the slope of the linear region in$\log J$ or$\ln J$ versus *E* can be used to evaluate the hopping behavior.

Among the four models, the Schottky and Poole–Frenkel plots show only partial quasi-linearity for the present PP-based films. However, for these two mechanisms, physically meaningful fitting should yield reasonable optical dielectric constants or related extracted parameters. In the present system, the fitted parameters are inconsistent with the low measured dielectric constants of the PP-based films (Figure 2a, e), indicating that neither Schottky emission nor Poole–Frenkel emission can be assigned as the dominant transport mechanism. In addition, the $\log(J)$-$\log(E)$ plots do not exhibit a sufficiently broad and stable region with a slope characteristic of trap-free SCLC, suggesting that SCLC is not the primary mechanism over the full measured field range. By comparison, the hopping analysis provides the most self-consistent description of the leakage-current data. This result is physically consistent with the electronic-structure analysis in Figure 3c, which indicates deeper electronic states in LSR107, and with the proposed localization of LSR107 in amorphous and interfacial regions where trap-assisted carrier transport is expected to dominate. Therefore, the leakage-current behavior of the present PP-based films is considered to be mainly governed by trap-assisted hopping-like conduction.


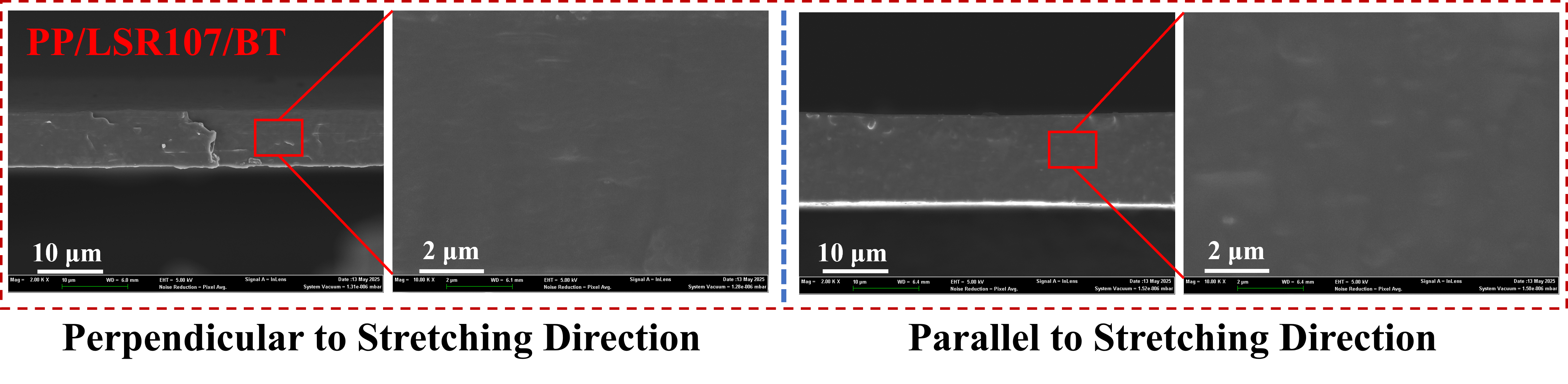


**Figure S15.** Cross-sectional SEM of a stretched PP/4.0 wt% LSR107/1.0 wt% BT composite film. The stretched composite shows a dense, void-free morphology after BT addition, confirming good filler–matrix integration at the mesoscale and effective stress transfer. This dense and void-free morphology helps mitigate local electric field concentrations that could otherwise lead to premature breakdown.


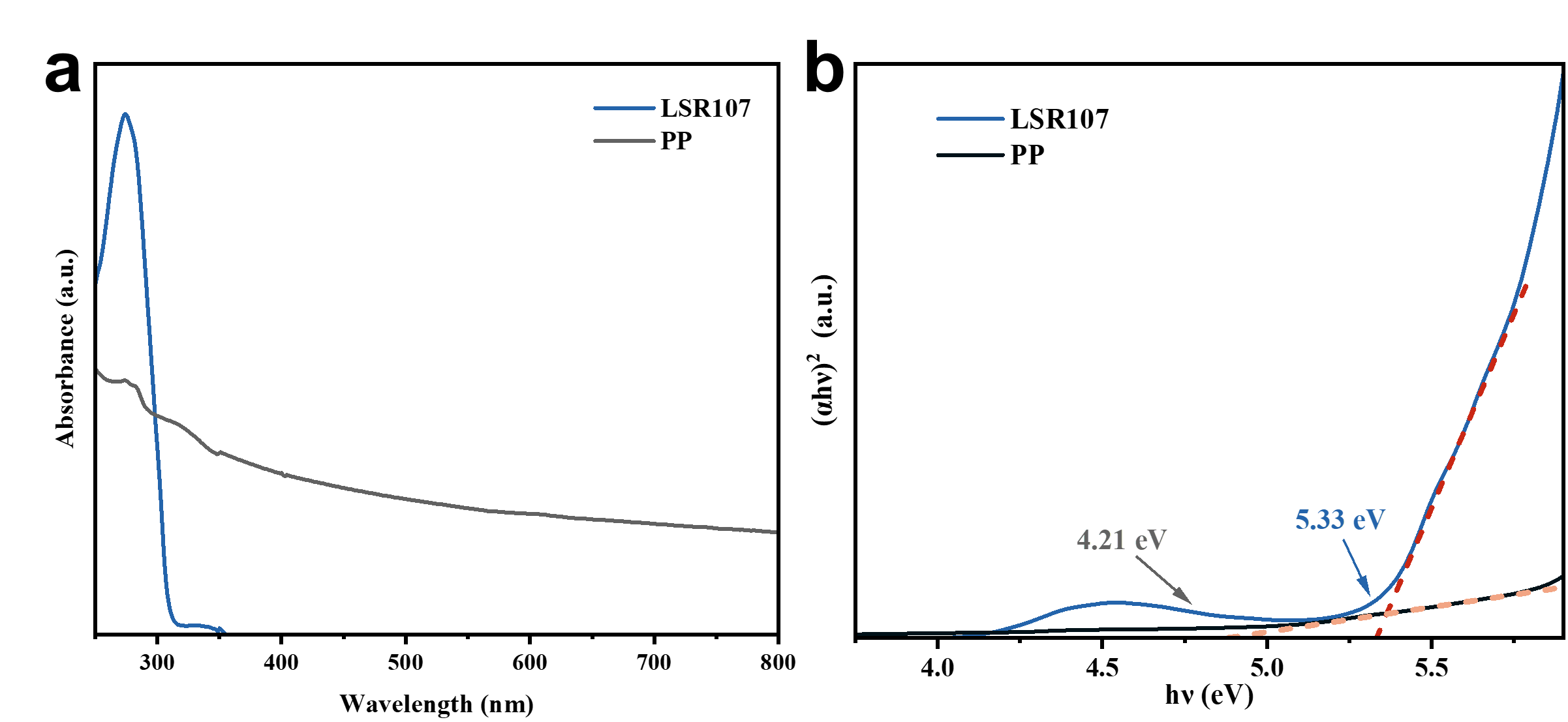


**Figure S16.** UV–Vis optical characterization of PP and LSR107, a) UV–Vis absorption spectra of neat PP and neat LSR107, b) tangent-line extrapolation method to determine the optical bandgap (Eg). The bandgap wavelength *λ_g_* is identified from the absorption edge, and the corresponding Eg is calculated as $Eg=hc/\lambda_{g}$ (where *h* is Planck’s constant and *c* is the speed of light).^[6-8]^


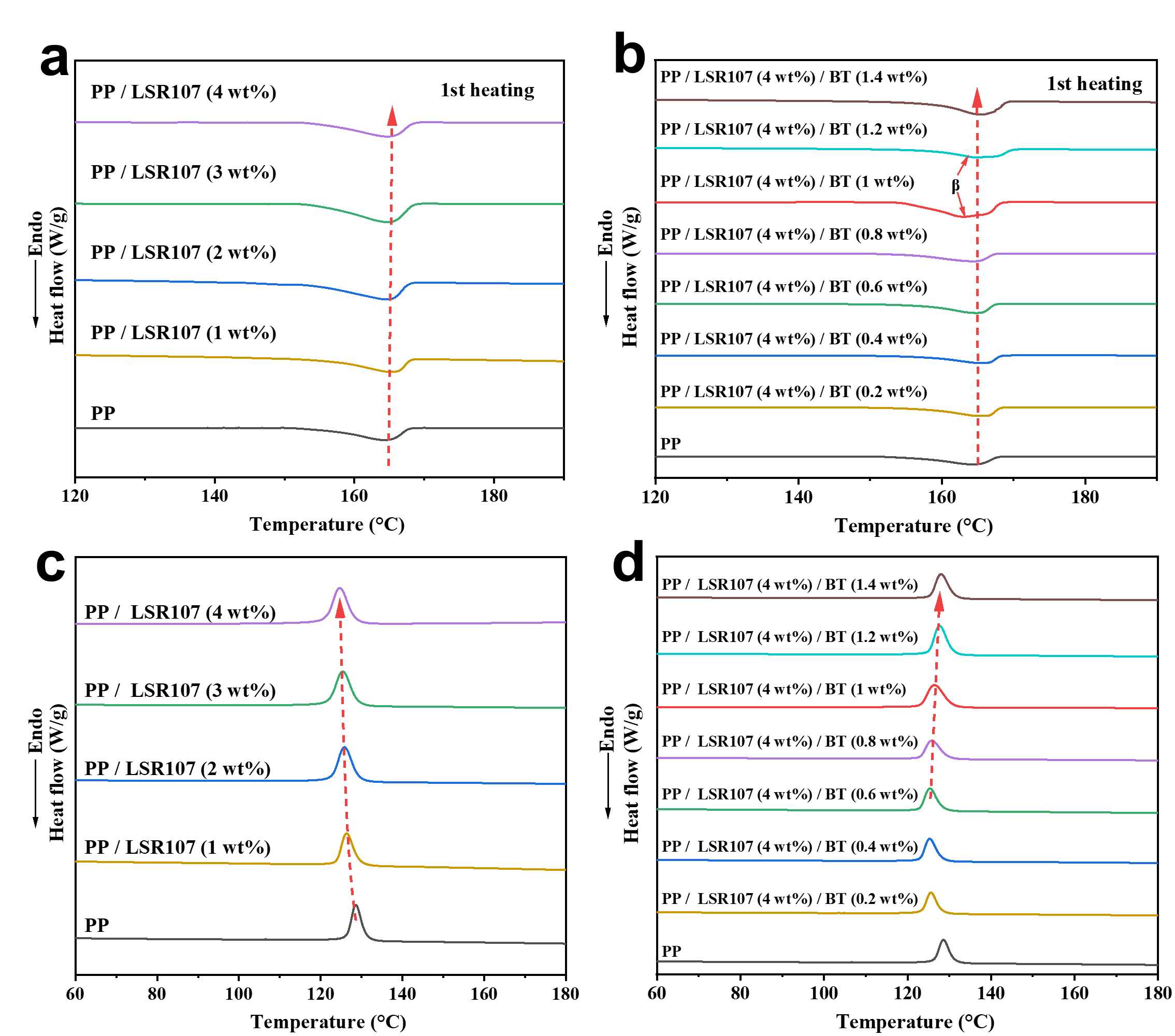


**Figure S17.** DSC thermal analysis, a) Melting curves for PP/LSR107, b) melting curves for PP/LSR107/BT, c) crystallization curves for PP/LSR107, d) crystallization curves for PP/LSR107/BT (heating/cooling rate 5 °C/min). The crystallization temperature (Tc) of PP decreases as LSR107 content increases, but is raised substantially by the inclusion of BT. For example, adding 4 wt% LSR107 shifts the PP crystallization peak to a temperature roughly 5–8 °C lower than that of neat PP, whereas introducing ≥1 wt% BT elevates the Tc by about 8–10 °C above PP/4 wt% LSR107. Meanwhile, the melting temperature (Tm) of PP in all composites remains in the 160–165 °C range.^[9-11]^


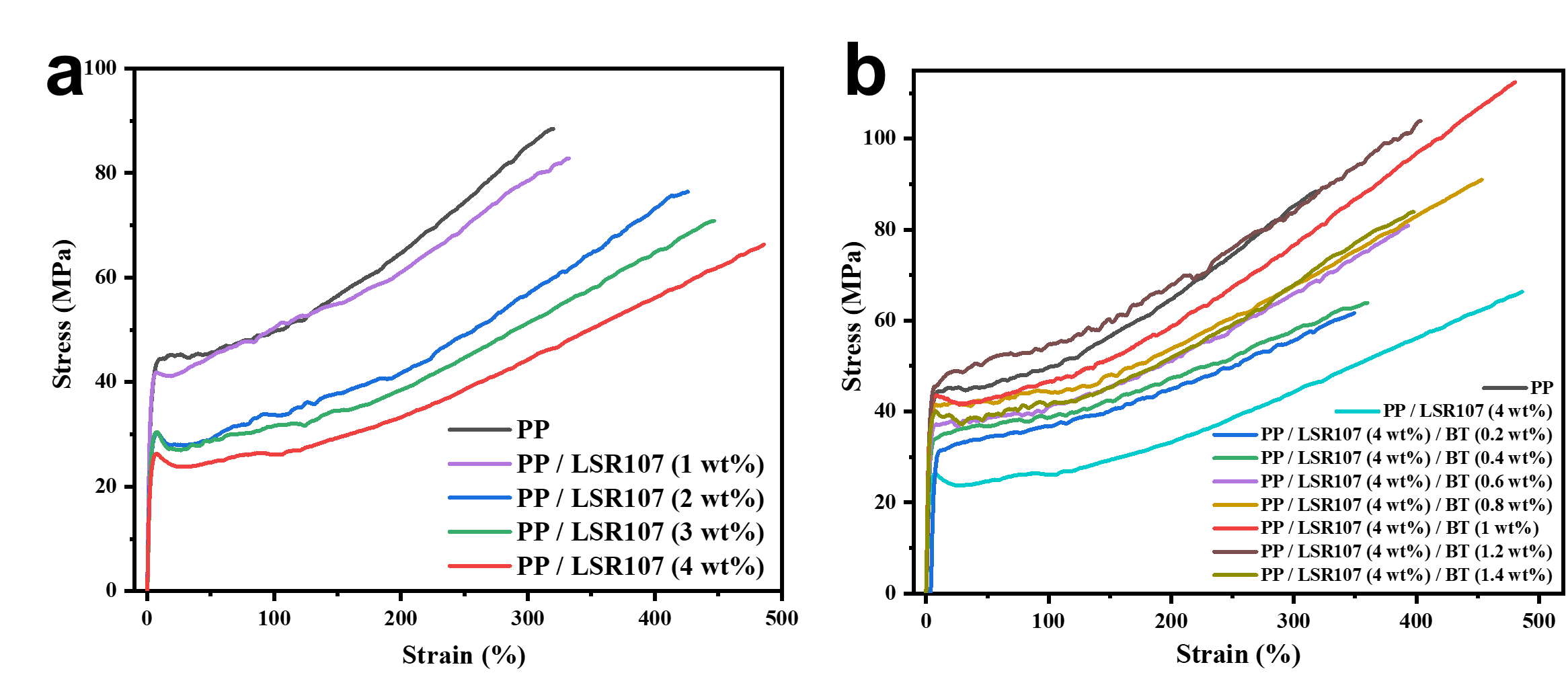


**Figure S18.** Tensile stress–strain behavior of modified PP films, a) PP/LSR107, b) PP/LSR107/BT (stretching speed = 50 mm/min). The inclusion of LSR107 (4 wt%) in PP greatly increases ductility but slightly reduces tensile strength. Adding a low fraction of BT (0.2–1.0 wt%) to the PP/LSR107 blend enhances the strength via stress distribution, whereas higher BT loadings (>1.2 wt%) induce particle agglomeration and defects that degrade the strength. The optimal BT content (~1.0 wt%) therefore balances toughness and rigidity, ensuring the mechanical integrity required for reliable dielectric film performance^[12-14]^.

**Supporting References**

1 W. Kim, M. Gu, P. Sriboriboon, et al., "Size dependent dielectric properties in BaTiO3 nanopowders for application of MLCC", *Applied Surface Science* 678 (2024): 161110, <https://doi.org/10.1016/j.apsusc.2024.161110>

2 M. A. Wahba, S. M. Yakout, A. M. Youssef, "Synergistic optical, dielectric and visible-light photocatalytic enhancement in Mo-modified BaTiO3 nanostructures", *Scientific Reports* 15 (2025): 37348, <https://doi.org/10.1038/s41598-025-22201-0>

3 Y. Liu, Y. Zhang, J. Wang, et al., "Ultrahigh capacitive energy storage through dendritic nanopolar design", *Science* 388 (2025): 211, <https://doi.org/10.1126/science.adt2703>

4 X. Li, B. Liu, J. Wang, et al., "High-temperature capacitive energy storage in polymer nanocomposites through nanoconfinement", *Nature Communications* 15 (2024): 6655, <https://doi.org/10.1038/s41467-024-51052-y>

5 G. Rui, J. Bernholc, S. Zhang, et al., "Dilute Nanocomposites: Tuning Polymer Chain Local Nanostructures to Enhance Dielectric Responses", *Advanced Materials* 36 (2024): 2311739, <https://doi.org/10.1002/adma.202311739>

6 T. Zhang, X. Chen, Y. Thakur, et al., "A highly scalable dielectric metamaterial with superior capacitor performance over a broad temperature", *Science Advances* 6 (2020): eaax6622, <https://doi.org/10.1126/sciadv.aax6622>

7 Q. Zhang, Z. Zhu, J. Zhu, et al., "High energy storage performances in multilayer composites via spatial structure design", *Composites Science and Technology* 270 (2025): 111286, <https://doi.org/10.1016/j.compscitech.2025.111286>

8 N. E. Stankova, P. A. Atanasov, N. N. Nedyalkov, et al., "fs- and ns-laser processing of polydimethylsiloxane (PDMS) elastomer: Comparative study", *Applied Surface Science* 336 (2015): 321, <https://doi.org/10.1016/j.apsusc.2014.12.121>

9 R. K. Mishra, S. Goel, I. Chianella, et al., "Graphene Nanoplatelets/Barium Titanate Polymer Nanocomposite Fibril: A Remanufactured Multifunctional Material with Unprecedented Electrical, Thermomechanical, and Electromagnetic Properties", *Advanced Sustainable Systems* 7 (2023): 2300177, <https://doi.org/10.1002/adsu.202300177>

10 Y. Wu, H. Zhao, N. Zhang, et al., "Improved energy storage properties of polypropylene-based composite dielectrics by introducing surface-charged BaTiO_3_@chitisan ultrafine constructions", *Journal of Materials Chemistry C* 12 (2024): 2993, <https://doi.org/10.1039/D3TC04180H>

11 K. Zhang, J. Yao, F. Zhu, et al., "Recent Advances in Preparation and Application of BOPP Film for Energy Storage and Dielectric Capacitors", *Molecules (Basel, Switzerland)* 30 (2025): 1596, <https://doi.org/10.3390/molecules30071596>

12 B. Wu, X. Zheng, W. Xu, et al., "β-Nucleated Polypropylene: Preparation, Nucleating Efficiency, Composite, and Future Prospects", *Polymers* 15 (2023): 3107, <https://doi.org/10.3390/polym15143107>

13 M.-S. Zheng, Y.-T. Zheng, J.-W. Zha, et al., "Improved dielectric, tensile and energy storage properties of surface rubberized BaTiO3/polypropylene nanocomposites", *Nano Energy* 48 (2018): 144, <https://doi.org/10.1016/j.nanoen.2018.03.049>

14 Q. Wu, J. Fang, M. Zheng, et al., "Morphology Evolution and Rheological Behaviors of PP/SR Thermoplastic Vulcanizate", *Polymers* 11 (2019): 175, <https://doi.org/10.3390/polym11010175>
